# Widespread circulation of West Nile and Usutu viruses in sedentary and migratory avifauna: A two-year study (2024–2025) of active surveillance in South of France

**DOI:** 10.1371/journal.pntd.0014397

**Published:** 2026-09-03

**Authors:** Rachel Beaubaton, Justine Revel, Laetitia Pigeyre, Alexandre Lepeule, Julien Joly, Christophe de Franceschi, Anne Charmantier, Benjamin Vollot, Yannick Simonin

**Affiliations:** 1 Pathogenesis and Control of Chronic and Emerging Infections (PCCEI), Université de Montpellier, Inserm, Montpellier, France; 2 CEFE, Univ Montpellier, CNRS, EPHE, IRD, Montpellier, France; 3 BV'Nat, Expert Indépendant, Aigues-Vives, France; Washington State University, UNITED STATES OF AMERICA

## Abstract

West Nile virus (WNV) and Usutu virus (USUV) are neurotropic Orthoflaviviruses sharing a similar enzootic transmission cycle primarily involving *Culex pipiens* mosquitoes as vectors and birds as amplifying hosts. First identified in Africa, both viruses established endemicity across Europe over the past two decades, most likely introduced and spread by migratory bird species along Mediterranean flyways. In avian species, infection outcomes range from subclinical to fatal neuroinvasive disease, varying by viral strain, host immunity, and species sensibility. Southern France emerges as a key hotspot for the circulation of these viruses, supported by diverse avian habitats conducive to year-round viral maintenance. This study investigated the RNA prevalence of WNV and USUV in more than 2500 sedentary and migratory wild birds from this region during 2024–2025 using molecular surveillance. Samples were collected using mists net and bird boxes, across multiple passerine and non-passerine taxa, spanning wetlands, urban fringes, and agricultural zones. Our analyses revealed widespread viral detection across diverse species, mainly among passerines such as great tits, house sparrows, and barn swallows with USUV detected at higher rates than WNV in both study years. Overall RNA prevalence was markedly higher in 2024 than in 2025, potentially reflecting climatic or ecological drivers. These results highlight the pivotal role of mixed avifauna in arbovirus dynamics within Mediterranean Europe and emphasize the necessity for integrated, year-round surveillance targeting high-risk species and habitats.

## Author summary

West Nile virus and Usutu virus are two closely related viruses spread by mosquitoes that mainly infect birds but can occasionally cause severe neurological disease in humans and horses. Southern France, and in particular the Camargue wetlands near Montpellier, is a long-recognised hotspot for both viruses. Over two years (2024–2025), we sampled more than 2,500 wild birds of 69 species in this region, testing cloacal swabs and droppings for viral RNA. Both viruses circulated widely, with Usutu virus detected roughly three times more often than West Nile virus, and overall circulation was higher in 2024 than in 2025 and more frequent in rural, wetland-associated sites. Our findings reinforce the value of active, non-invasive avian surveillance as a complement to existing human and veterinary case reporting for these viruses in Europe.

## Introduction

West Nile virus (WNV) and Usutu virus (USUV), closely related members of the Japanese encephalitis virus serocomplex, are arboviruses of the genus *Orthoflavivirus* from the *Flaviviridae* family. They were first identified and isolated in Africa, WNV in 1937 in Uganda in the West Nile district [1] and USUV in 1959 in South Africa near the Usutu River [2]. They share similar biological traits, transmission cycle and epidemiology [3]. *Culex pipiens* mosquitoes, which are primarily ornithophilic, can transmit WNV and USUV from birds, considered the main amplifying hosts, to various mammalian species, including humans, horses, dogs, bats, wild boars, and several ruminants such as deers, cattles, and sheeps, which are presumed to be dead-end hosts [4,5]. Most human infections with WNV and USUV are asymptomatic, but when symptoms occur, they are usually mild, including fever, headache, rash, or muscle and joint pain. Severe neurological complications, including encephalitis or meningitis, may occasionally occur. Current evidence suggests that WNV is more virulent in humans than USUV whereas the opposite pattern is observed in birds [1,5–8]. Approximately 30 neuroinvasive USUV cases of varying severity in humans have been reported in Europe since 2016, while WNV occasions each year several hundred human cases and dozens of deaths across Europe [4,6]. USUV is classified into eight distinct genetic lineages, grouped into African (Africa 1/2/3) and European (Europe 1/2/3/4/5) clusters, with virulence potentially varying between lineages [9–11]. Among the nine known WNV lineages, infections are primarily caused by lineages 1 and 2, with lineage 2 notably contributing to the recent rise in severe neuroinvasive human cases in Europe [12,13].

The epidemiology of WNV and USUV has changed considerably over the past two decades in Europe, with repeated epizootic events contributing to their progressive endemization. USUV was first associated with significant avian mortality in Europe in 1996, affecting blackbird (*Turdus merula*) populations in Italy [6]. A notable amplification event occurred in 2018, when outbreaks of both viruses affected several European countries [4,14–16]. USUV was associated with high mortality in multiple bird species, particularly blackbirds (*Turdus merula*), magpies (*Pica pica*) and Strigiformes. During the same season, WNV activity also increased in avian hosts, with initial detections in resident wild and captive birds in Germany and widespread evidence of WNV infection across multiple bird species and carcasses [4,16–20]. Since then, both viruses have become widely established across Central and Western Europe, causing recurrent avian outbreaks and demonstrating a progressive northward expansion of their distribution range [21–29]. The endemization of WNV and USUV in Europe is thought to be influenced by repeated introductions mediated by long-distance migratory birds along major Afro-Palearctic flyways, followed by local amplification within resident bird–mosquito transmission cycles [30]. This applies to tits (*Paridae*), sparrows (*Passeridae*) and pigeons (*Columbidae*), which are resident passerines widely distributed across the continent and identified in the literature as being susceptible to both WNV and USUV [7,31]. Migratory species proposed as potential contributors to viral dispersal into new European regions include the common kestrel (*Falco tinnunculus*) and the lesser whitethroat (*Curruca curruca*), both of which migrate between Africa and Europe [4–6,9]. The first recorded entry from Africa into Spain aligns with the East Atlantic migratory pathway, while its introduction to Central Europe appears to follow the Black Sea/Mediterranean route [6]. Comparable introduction patterns have been described for WNV, with multiple lineage exchanges between Africa and Europe along these migratory corridors [32].

In France, WNV and USUV infections have mainly been reported in the south, especially in the city of Montpellier. The nearby Camargue Regional Nature Park and surrounding wetlands provide an optimal environment for arbovirus maintenance, with dense avian populations, proximity to major migratory routes, and high mosquito vector activity [33]. Infections have been reported in humans [34], mosquitoes [33,35], birds [36,37], dogs, horses [33] and in several species of the Montpellier zoological park [25,37]. Notably, the first human USUV infection in France was reported in 2016, in a patient from Montpellier presenting idiopathic facial paralysis [34]. Serological screening in the same region revealed that 3% of blood donors had antibodies against USUV, suggesting local viral circulation [33]. Moreover, the first human case of WNV in Europe was identified in the Camargue in 1962, and in 2024 12 human cases of WNV infection were reported in our study area [38].

WNV and USUV primarily circulate in wild birds and follow complex transmission cycles, making surveillance of both resident and migratory species essential to understand their dynamics. Based on this background, the specific objectives of this study were to assess the extent of WNV and USUV circulation in the avifauna of the Montpellier-Camargue region; to identify, within the limits of opportunistic field sampling, the species presenting the highest RNA detection prevalence; to identify additional bird species naturally infected with WNV or USUV; to document the temporal sequence of detections across the multi-month sampling period of each year; and to describe, through exploratory comparisons, how detection patterns were distributed between sampled migratory and resident birds. More than 2500 avian samples, across 69 species, were sampled between May–December 2024 and February–October 2025 at different sites (based on avian density, wetlands proximity, and vector density). Cloacal swabs and droppings were analyzed using a WNV/USUV RT-qPCR TaqMan duplex assay. Our findings reveal a markedly high circulation of both viruses in avian hosts in 2024, followed by lower activity in 2025. This circulation occurred early in the season, emerging well before the detection of cases in humans and horses, and preceding virus detection in mosquito vectors. Over the two-year period, USUV exhibited nearly three times the molecular prevalence of WNV across both resident and migratory bird species, with limited co‑infections. Several species showed particularly high RNA prevalence for both viruses, most notably great tits (*Parus major)*, house sparrows (*Passer domesticus)*, and barn swallows (*Hirundo rustica)*. Viral activity was detected across urban, peri‑urban, and rural habitats, underscoring the broad ecological footprint of both pathogens. Together, these patterns highlight the central role of avifauna in the spatial dynamics of WNV and USUV.

## Methods

### Ethics statement

All avian captures, handling, and sampling procedures were conducted in accordance with established ethical guidelines for wildlife research and were approved by the CRBPO, under the authority of the French National Museum of Natural History (Muséum national d’Histoire naturelle, MNHN; programme number 1331, Bird ringing permit No. 1820). For studies conducted by CEFE, authorization number F 3417211 was issued in February 2024. No animals were captured or sampled exclusively for the purpose of this study. Handling time per individual typically did not exceed a few minutes, consisting of standard ringing procedures together with cloacal swab and dropping collection; mist nets were checked every 30 minutes throughout to minimise stress and injury risk, as described above. No sampling-related mortality or adverse events were recorded during the study. Ringing and sampling activities on wild birds in France are conducted under the national CRBPO authorisation framework, which provides the equivalent ethical and welfare oversight for this type of fieldwork in the absence of a dedicated institutional animal care and use committee for wildlife ringing studies. Laboratory processing of samples followed standard biosafety practices for arbovirus handling.

### Survey area

**Fig 1:**
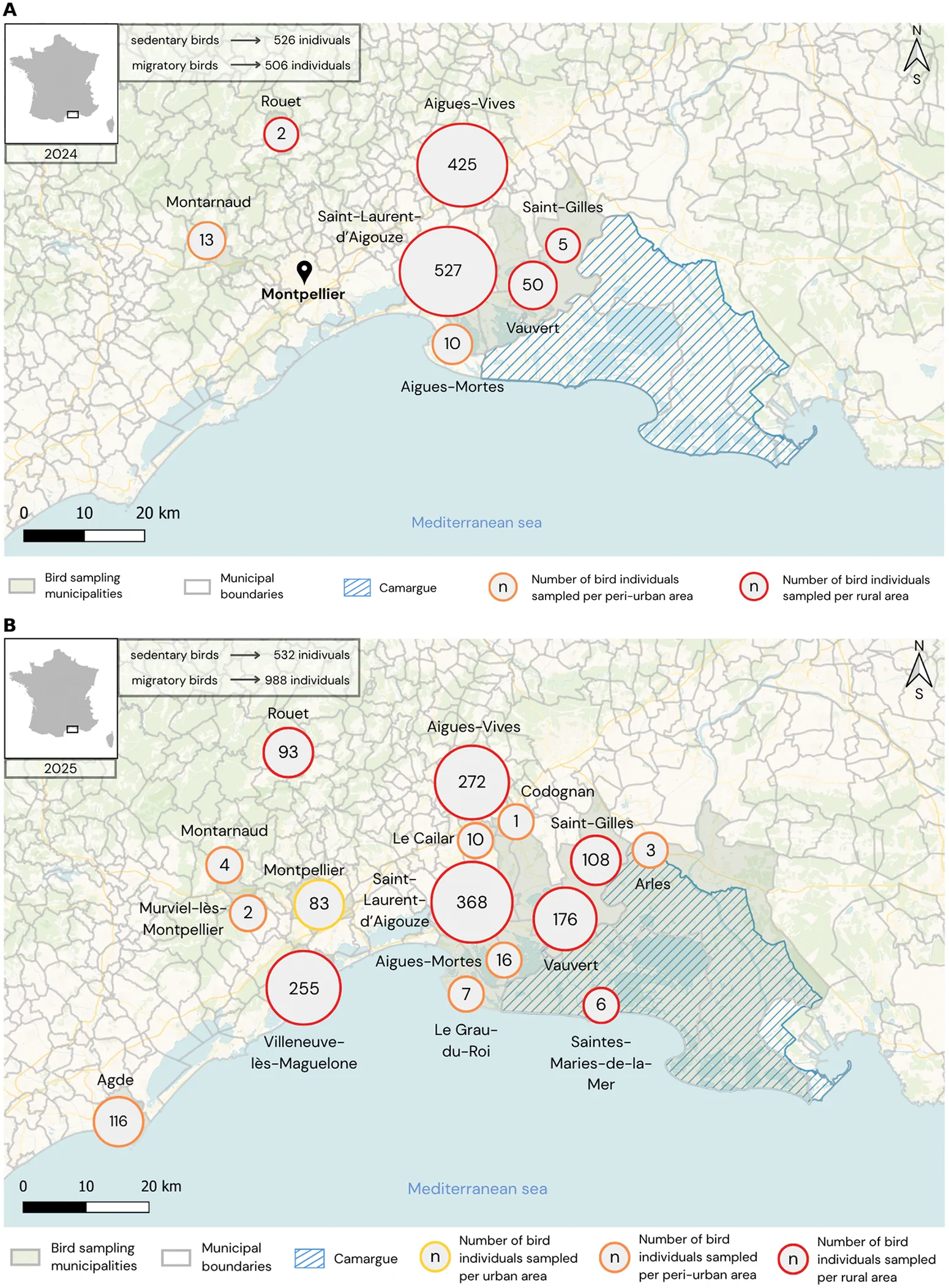
Study area and avian sampling locations in southern France during the 2024-2025 surveillance campaigns. Sites were selected based on bird density and proximity to wetlands and mosquito breeding habitats. Each circle is centred on a sampling municipality; its size and inset number indicate the number of individual birds captured there, and its outline colour indicates the locality type as defined in the Methods (yellow: urban; orange: peri-urban; red: rural). Sampling municipalities are shaded in beige; the hatched area delineates the Camargue wetland complex. A scale bar (0–20 km) and north arrow are provided. The inset map in the top-left corner situates the study area within France and summarises the total numbers of sedentary and migratory individuals sampled that year. Base map: CARTO Voyager No Labels (https://carto.com/basemaps/); map tiles CARTO, licensed CC BY 3.0 (https://creativecommons.org/licenses/by/3.0/); map data OpenStreetMap contributors, licensed under the Open Database License (ODbL) (https://www.openstreetmap.org/copyright; licence text: https://opendatacommons.org/licenses/odbl/1-0/index.html). Overlay layers: French municipal boundaries and Parcs Naturels Régionaux (PNR) boundaries, derived from OpenStreetMap and distributed via data.gouv.fr under ODbL. https://www.data.gouv.fr/datasets/parcs-naturels-regionaux-pnr-france-metropolitaine; https://www.data.gouv.fr/datasets/decoupage-administratif-communal-francais-issu-d-openstreetmap.

The study was conducted in southern France, within the Mediterranean basin, and focused on a geographically continuous region encompassing two main areas: the Camargue wetland and the urban and peri‑urban surroundings of Montpellier, located in the eastern part of the Occitanie region. This region is characterized by a warm-summer Mediterranean climate as defined by the Köppen–Geiger classification [39]. The Camargue represents one of the largest natural wetland complexes in Western Europe and is situated between the two arms of the Rhône River and the Mediterranean coastline. This study area is mainly located in the Petite Camargue in the Occitanie region, corresponding to the western part of the Camargue. The flat and low landscape is shaped by an extensive network of cultures, canals, ditches, and wetlands that maintain high levels of humidity even in otherwise dry areas. These environmental conditions support both diverse avian communities and dense mosquito populations, creating a favorable ecological context for arbovirus circulation [40]. Birds were sampled in lowland areas, where the WNV and USUV vector *Culex pipiens* is most abundant. In 2024, birds were sampled in seven different municipalities (Fig 1A): 43°39'45.8"N 3°39'52.8"E (Montarnaud, 34163); 43°49'25.1"N 3°47'43.5"E (Rouet, 34380); 43°35'36.8"N 4°11'57.2"E (Tour Carbonnière, Saint-Laurent-d’Aigouze, 30220); 43°42'34.8"N 4°12'12.7"E (Carrière, Aigues-Vives, 30670); 43°35'04.4"N 4°12'27.6"E (Aigues-Mortes, 30220); 43°36'20.7"N 4°20'13.9"E (Centre de découverte du Scamandre, Vauvert, 30600); 43°38'42.4"N 4°24'14.0"E (Espeyran, Saint-Gilles, 30800). In 2025, 16 different municipalities were used for sampling (Fig 1B): 43°39'45.8"N 3°39'52.8"E (Montarnaud, 34163); 43°49'28.6"N 3°47'39.2"E (Rouet, 34380); 43°35'36.8"N 4°11'57.2"E (Tour Carbonnière, Saint-Laurent-d’Aigouze, 30220); 43°42'34.8"N 4°12'12.7"E (Carrière, Aigues-Vives, 30670); 43°35'04.4"N 4°12'27.6"E (Aigues-Mortes, 30220); 43°36'20.7"N 4°20'13.9"E (Centre de découverte du Scamandre, Vauvert, 30600); 43°38'42.4"N 4°24'14.0"E (Espeyran, Saint-Gilles, 30800); 43°38'20.0"N 3°51'42.1"E (CEFE, Montpellier, 34090); 43°36'30.5"N 3°42'36.6"E (Murviel-lès-Montpellier, 34570); 43°18'43.1"N 3°31'48.2"E (Réserve Naturelle du Bagnas, Agde, 34300); 43°31'02.6"N 3°50'04.8"E (Villeneuve-lès-Maguelone, 34750); 43°33'04.7"N 4°06'54.7"E (Grau-du-Roi, 30240); 43°28'41.4"N 4°23'38.5"E (Saintes-Maries-de-la-Mer, 13460); 43°39'45.99"N 4°28'47.87"E (Arles, 13200); 43°42'08.0"N 4°14'18.2"E (Le Cailar, 30740); 43°43'23.6"N 4°13'53.7"E (Codognan, 30920). These sites were selected based on bird density, proximity to wetlands and/or the density of mosquito vectors. Sampling sites were classified a priori into three locality types according to their dominant surrounding land-use context. Urban sites corresponded to continuous built-up areas and included Montpellier. Peri-urban sites corresponded to transitional areas at the interface between built-up environments and agricultural or natural habitats and included Agde, Aigues-Mortes, Arles, Codognan, Le Cailar, Le Grau-du-Roi, Montarnaud and Murviel-lès-Montpellier. Rural sites corresponded to areas dominated by wetlands, agricultural land or natural habitats with low building density and included Aigues-Vives, Rouet, Saintes-Maries-de-la-Mer, Saint-Gilles, Saint-Laurent-d’Aigouze, Vauvert and Villeneuve-lès-Maguelone.

### Avian sampling and definition of analytical units

In 2024, 1110 biological samples were collected from 15 May to 2 December, including 1039 cloacal swabs and 71 droppings; both sample types were available for the same bird during 35 capture events. In 2025, 1773 biological samples were collected from 23 February to 28 October, including 1507 cloacal swabs and 266 droppings; both sample types were available for the same bird during 187 capture events. When both a cloacal swab and a dropping were available for the same bird at the same capture event, the results from the two sample types were combined to assign a single detection status for each virus at that capture: a capture event was considered positive if at least one available sample tested positive, and negative if all available samples tested negative. After combining paired samples in this way, 1075 capture events were retained in 2024 and 1586 in 2025, for a total of 2661 capture events across both years. Capture-event-level data were retained only for the description of monthly detection patterns and for the longitudinal analysis of recaptured individuals. When the same ringed bird was captured on multiple occasions within a given year, capture-specific results were aggregated to obtain a single annual detection status per individual: an individual was considered positive if at least one capture event tested positive, and negative if all capture events tested negative. After repeated within-year captures were combined, 1032 individual birds were retained for the 2024 campaign and 1520 for the 2025 campaign, corresponding to 2552 year-specific individual records. 19 individuals were captured in both years; these birds were counted once in each year-specific descriptive summary but only once when the two campaigns were combined, giving a total of 2533 unique individuals across the study period. The unique individual bird was therefore the unit of analysis for the main prevalence estimates and statistical analyses

### Field capture protocol and biological sample collection procedures

Wild birds were captured using mist nets installed in habitats visited by the target species, particularly along flight corridors, forest edges, and near feeding areas. As the objective of this study was not to assess population trends, sampling effort was not standardized across sessions. For each session, the surface area covered, number of net-hours, and duration of net opening were recorded. Mist nets with an appropriate mesh size were selected according to the expected size of species present in the study area. Nets were mounted vertically between two poles and operated depending on weather conditions. They were checked at 30-minute intervals to minimize handling time, reduce stress, and limit the risk of injury to captured individuals. Captured birds were carefully removed from the nets and the species identified. Individuals were fitted with uniquely numbered metal leg bands provided by the Centre de Recherches sur la Biologie des Populations d’Oiseaux (CRBPO (Centre for Research on the Biology of Bird Populations), French Museum of Natural History). All birds were released at their site of capture immediately after processing. Standard morphometric measurements were obtained from a subset of individuals, including body mass, wing length (flattened and straightened), tarsus length, and bill length. These checks were mandatory prior to sampling as they enable ornithologists to verify that the bird is in good health, but they were not used in our analyses. A visual clinical examination was performed on each individual. Cloacal swabs and fecal samples were collected following the standardized protocol described by Knutie et al. [41].

### RNA extraction, qRT-PCR and sequencing

RNA extraction was carried out from 140 µL of cloacal swabs and droppings using the QIAamp Viral RNA Mini Kit (Qiagen), according to the manufacturer’s instructions. For cloacal swabs, 140 µL of the viral transport medium (VTM) in which the swab was immersed was directly processed. For droppings, samples were ground in 140 µL of PBS 1x prior to extraction. At the end of the procedure, 50 µL of RNA eluate was recovered for each sample before proceeding to a one‑step duplex TaqMan qRT‑PCR assay. After extraction, 5 μL of each RNA sample was tested with SuperScript IV (Invitrogen), WNV primers (Forward: CCTGTGTGAGCTGACAAACTTAGT and Reverse: GCGTTTTAGCATATTGACAGCC) with a probe (5’CY5-CCTGGTTTCTTAGACATCGAGATCT-3’BHQ2), and USUV primers (Forward: AAAAATGTACGCGGATGACACA and Reverse: TTTGGCCTCGTTGTCAAGATC) with a probe (5'FAM-CGGCTGGGACACCCGGATAACC-3’TAMRA). Cycling conditions were as follows: reverse transcription at 50°C for 15 minutes; inactivation of reverse transcriptase and activation of Taq polymerase at 95°C for 2 minutes; followed by 50 cycles of denaturation at 95°C for 15 seconds and elongation at 60°C for 1 minute. All reactions were performed on a LightCycler 480 (Roche).

Samples were considered positive for a Ct value up to and including 40, with regular verification by sequencing of the corresponding TaqMan PCR amplicons. Samples with Ct values between 40 and 45 were considered doubtful and were systematically checked by amplicon sequencing to confirm or rule out positivity. For RT-qPCR-positive samples with Ct values below 35, a pan-orthoflavivirus PCR targeting a longer fragment of the envelope gene (approximately 250 base pairs) was performed to generate larger amplicons suitable for downstream sequencing [42]. In parallel, virus-specific one-step RT-PCR assays were performed using the complete sets of eight overlapping primer pairs developed for WNV and USUV whole-genome amplification, generating amplicons of approximately 1–2 kb as previously described [43]. Amplicons obtained with both approaches were purified using the NucleoSpin Gel and PCR Clean-up Kit and were subsequently submitted for sequencing. Sanger sequencing was performed by GENEWIZ (Azenta Life Sciences) using 20 µL of purified amplicons and the corresponding amplification primer.

### Phylogenetic analyses

For WNV thirty-three reference nucleotide sequences of the virus were aligned with four sequences from avian samples and three sequences from mosquitoes. The alignment was complemented by a sequence from Japanese encephalitis virus (JEV). For USUV fifty reference nucleotide sequences of the virus were aligned with two sequences from this study. The alignment was complemented by a sequence from WNV. The tool used for the alignment was MAFFT version 7.505 (_). The MAFFT output data were cleaned and trimmed using TrimAl version 1.5.rev1 (_) to optimize tree construction. A maximum likelihood phylogenetic tree was constructed with IQ-TREE version 3.0.1 (_), with automatic detection of the most appropriate model according to the Bayesian Information Criterion (BIC). Node support was assessed jointly using ultrafast bootstrap approximation (UFBoot2, 1,000 replicates) and the Shimodaira-Hasegawa-like approximate likelihood ratio test (SH-aLRT, 1,000 replicates), computed together in IQ-TREE 3.0.1 (options -bb 1000 -alrt 1000). The final tree was visualized and annotated with the online version iTOL version 7.5 (_) and rerooted with the JEV or WNV sequence. The sequences generated in this study have been deposited in GenBank as follows: USUV avian samples: AX24421 (PZ724027) and AX1309 (PZ724028); WNV avian samples: AX76078 (PZ724029), AX76022 (PZ724030), AX76014 (PZ724031) and JA78956 (PZ724032); WNV mosquito samples: WNV-MX-MTP-TAP-France/2025-250729 (OZ263593) and WNV-MX-SJV-CAN-France/2025-250910 (OZ263594). All raw datasets, metadata, sequence alignments, phylogenetic trees (annotated with both bootstrap and SH-aLRT support values), and analysis scripts used in this study are publicly available (https://github.com/Simonin3488/wnv-usuv-camargue-phylo) and have been archived on Zenodo ((https://zenodo.org/records/21340737).

### Statistical analysis

Overall RNA prevalence was calculated as the proportion of positive birds among the total number of birds tested and was reported with two-sided 95% confidence intervals (95% CI) using the Wilson method. Throughout this manuscript, the term prevalence refers strictly to the proportion of birds testing positive for viral RNA by RT-qPCR (i.e., a molecular detection rate) and does not imply confirmed active infection, viraemia, or infectiousness, which would require complementary virological evidence. The concordance between WNV and USUV infection status was assessed using McNemar’s test, first on the overall study population and then stratified according to migration pattern. Differences in the frequency of co-infections between 2024 and 2025 were evaluated using Fisher’s exact test.

Factors associated with WNV and USUV infections were investigated separately using generalized linear mixed models (GLMMs) with a binomial distribution and logit link function. Species was included as a random effect. Adjusted odds ratios (OR) and their 95% confidence intervals (95% CI) were estimated. Model building was performed using a two-step variable selection procedure. First, each explanatory variable was evaluated individually using a univariable mixed-effects logistic regression model including species as a random effect. Variables with a p-value < 0.20 were retained for inclusion in the initial multivariable model. A backward stepwise selection procedure was then applied. At each step, the contribution of each variable was assessed using likelihood ratio tests (LRT), and variables that did not significantly improve model fit were sequentially removed until the final model was obtained. The adequacy of the final models was assessed using simulated residuals generated with the DHARMa package, including evaluation of model specification, dispersion, and outliers. Model singularity was also assessed. Multicollinearity among explanatory variables was evaluated using variance inflation factors (VIF). Model fit was further quantified using marginal and conditional R^2^ and the intraclass correlation coefficient (ICC) was calculated to assess the contribution of the species-level random effect. All statistical analyses were performed using R software (R Core Team, 2025) within the RStudio environment (version 4.5.1, 2025).

## Results

### Study areas and sampling strategies

Over the two-year study period, 2533 unique wild birds representing 69 species across eight taxonomic orders were sampled in urban, peri-urban, and rural areas. When considered separately by year, the sampling campaigns comprised 1032 individual birds in 2024 and 1520 in 2025, corresponding to 2552 year-specific individual records including 19 birds that were captured in both years (S1 Table; Fig 1).

In 2024, the sampled avifauna represented 54 species across seven orders. Among these species, 25 were sedentary species native to southern France, comprising 526 individuals, while the remaining 29 were migratory, comprising 506 individuals (S1 Table). In 2025, the sampled avifauna represented 60 species across six taxonomic orders, evenly distributed between sedentary (n = 30; 532 individuals) and migratory species (n = 30; 988 individuals). Compared with the 2024 campaign, 9 species were not sampled (*Actitis hypoleucos, Corvus corone, Ixobrychus minutus, Lanius collurio, Larus michahellis, Oriolus oriolus, Phylloscopus collybita tristis, Turdus philomelos, Upupa epops*), whereas 15 new species were sampled (*Acrocephalus paludicola, Caprimulgus europaeus, Certhia brachydactyla, Cisticola juncidis, Coccothraustes coccothraustes, Dryobates minor, Emberiza cirlus, Gallinago gallinago, Locustella naevia, Oenanthe oenanthe, Regulus ignicapilla, Saxicola rubetra, Strix aluco, Sylvia borin, Turdus merula*) (S1 Table).

**Fig 2:**
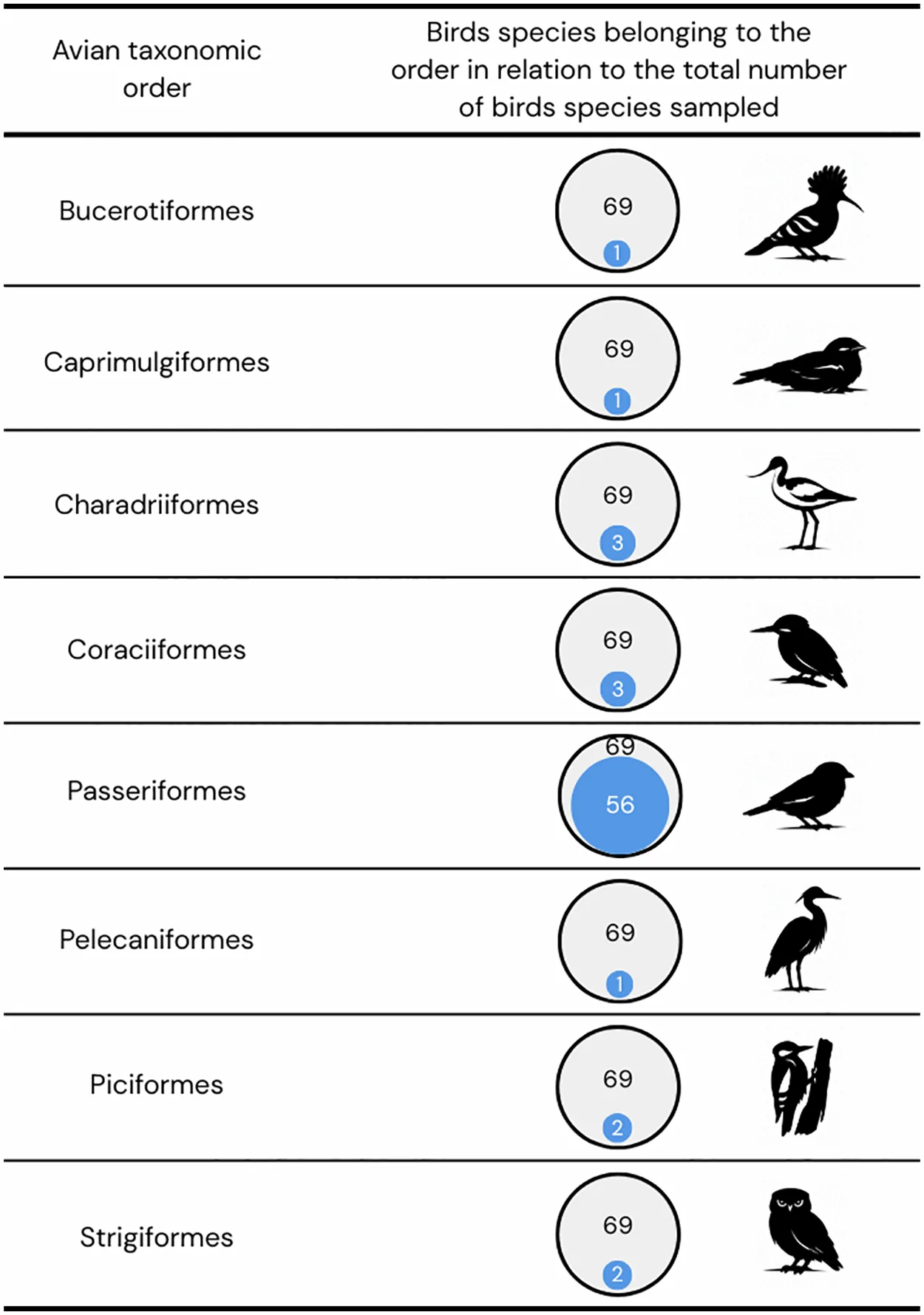
Proportion of avian taxonomic orders sampled in 2024 and 2025. Each row represents a bird taxonomic order. The grey circle indicates the total number of species sampled (n = 69, all orders combined). The blue circle represents the number of sampled species belonging to the focal order. Silhouettes on the right illustrate a representative species for each order. Circle size in this panel reflects species richness (the number of distinct species recorded) within each taxonomic order, per-species sample sizes and RNA prevalence estimates are provided in S1 Table. Silhouette illustrations generated using OpenAI, GPT-5.5, image generation and assembled by the authors (https://openai.com/policies/terms-of-use/).

Across both years, Passeriformes constituted the largest proportion of species sampled (n = 56, 81.2%), with major contributions from barn swallow (*Hirundo rustica*) (n = 654, 25.8%), common reed warbler (*Acrocephalus scirpaceus*) (n = 223, 8.8%), great tit (*Parus major)* (n = 176, 6.9%) and house sparrow (*Passer domesticus)* (n = 149, 5.9%) (Fig 2). The remaining 18.8% of species were distributed across seven other orders, including Bucerotiformes, Caprimulgiformes, and Pelecaniformes as the most underrepresented, each being represented by a single species. Cloacal swabs and/or fecal samples were collected from each bird according to sampling opportunities. All samples were subsequently subjected to molecular analysis for the detection of USUV and WNV RNA.

**Fig 3:**
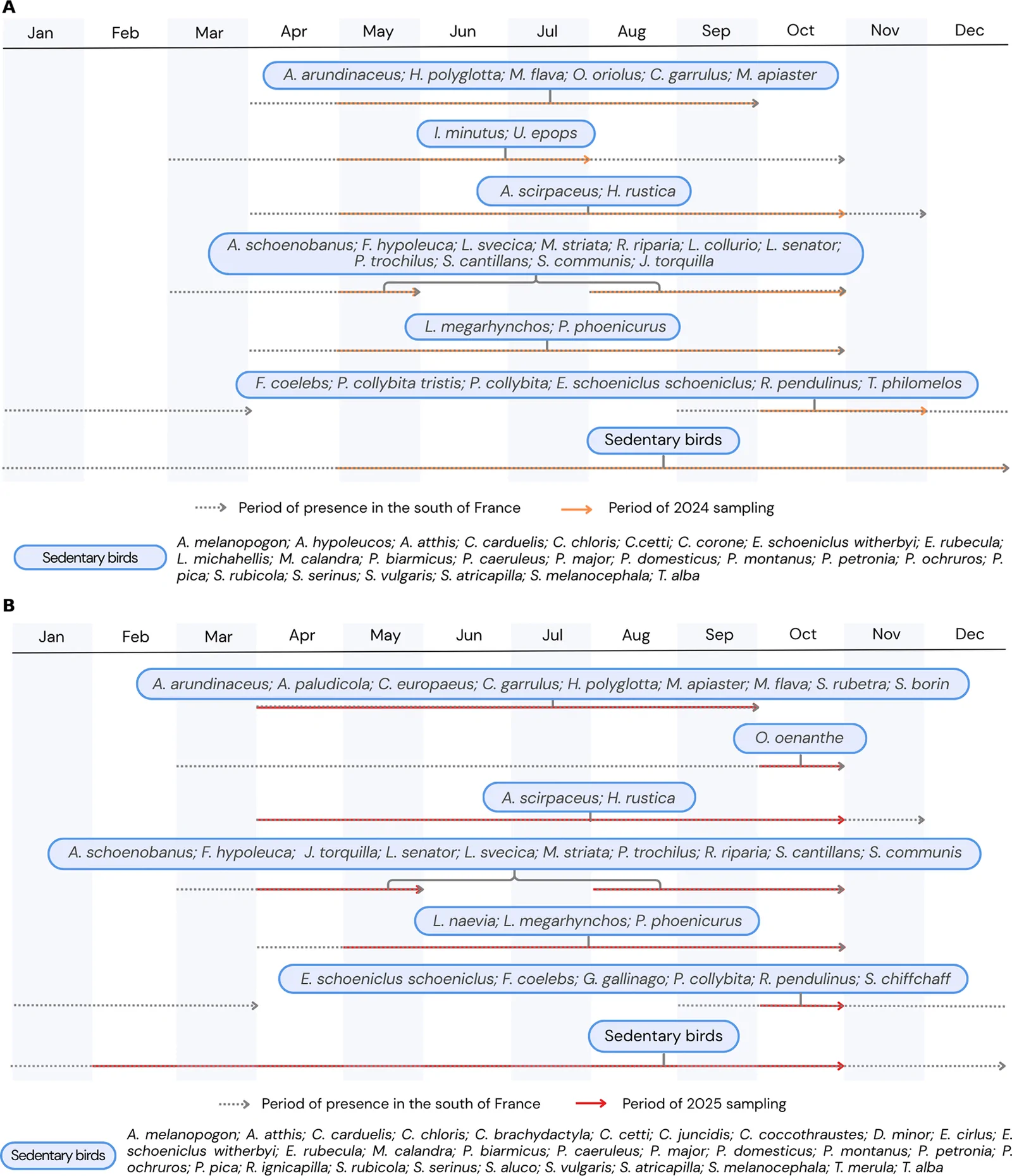
Annual chronological timelines of the sampling periods for bird species collected in 2024 (A) and 2025 (B), based on their periods of presence in the Camargue region. The horizontal span indicates the period during which the species was recorded and available for sampling in the study area, based on capture dates.

Sedentary bird species are present in the study area throughout the year and are therefore potentially available for sampling across all seasons. In contrast, migratory species exhibit heterogeneous patterns of presence (Fig 3). Several migratory species are known to use the Camargue area as a breeding and resting site and typically remain in the region for several months between spring and autumn, resulting in prolonged local presence during the sampling period. For example, the common reed warbler (*Acrocephalus scirpaceus*), the European bee-eater (*Merops apiaster*), the barn swallow (*Hirundo rustica*) and the great reed warbler (*Acrocephalus arundinaceus*) arrive in spring to breed and remain throughout summer before departing in autumn. Other migratory species are mainly present during shorter stopover phases associated with spring or autumn migration, leading to more limited temporal windows of exposure. Examples include various species of warblers such as the sedge warbler (*Acrocephalus schoenobaenus)* or the willow warbler (*Phylloscopus trochilus*), which transit through the Camargue during their migration and are typically observed only during these seasonal windows. By contrast, truly sedentary species, such as the Eurasian blue tit (*Cyanistes caeruleus*), the great tit (*Parus major*), the moustached warbler (*Acrocephalus melanopogon*), or the house sparrow (*Passer domesticus*), are recorded throughout the year, consistent with their resident status in the local habitats. These differences in residency status and duration of presence align with the known migratory ecology of the species involved and may influence their exposure to locally circulating arboviruses, with prolonged residents having a longer potential contact period with virus vectors compared to short-distance or passage migrants.

### Circulation of USUV/WNV in avifauna

**Fig 4:**
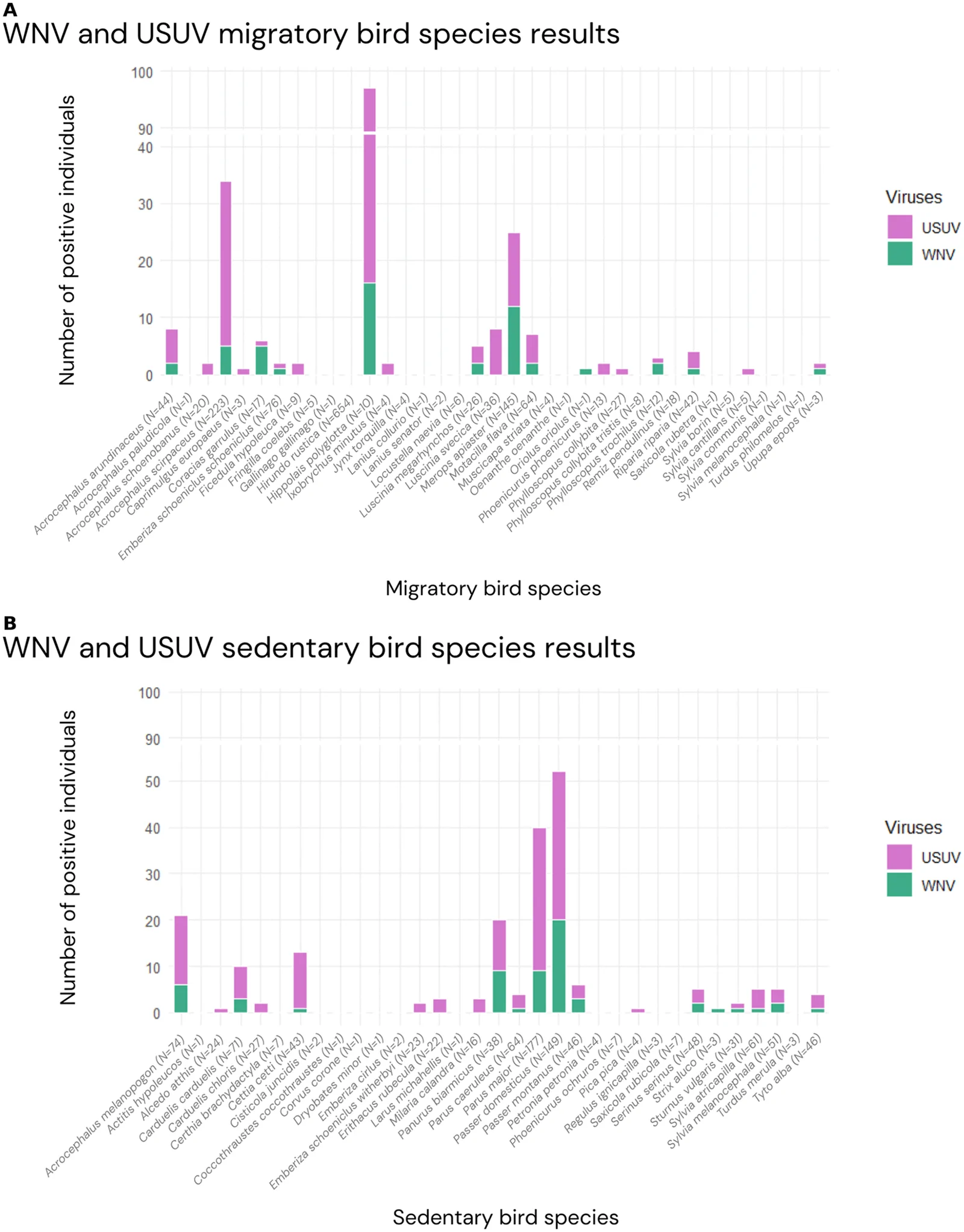
WNV and USUV positive detections by bird species in 2024 and 2025, stratified by migratory behavior. Number of individuals testing positive for West Nile virus (green) and Usutu virus (purple) among migratory (A) and sedentary (B) bird species on both years. Sample sizes for each species are indicated in parentheses along the x‑axis. Bars show the absolute number of individuals testing positive for West Nile virus (green) or Usutu virus (purple) by RT-qPCR, for migratory (A) and sedentary (B) species, with both study years pooled within each species bar. The number in parentheses after each species name is the total number of individuals of that species tested across both years, corresponding to the denominator used to calculate the RNA prevalence estimates reported in the Results and in S1 Table. Species-level prevalence with 95% confidence intervals is provided in S1 Table.

The distribution of USUV and WNV molecular detections across all sampled avifauna, considering 2024 and 2025 together, showed a significant difference in detection frequencies between the two viruses (McNemar’s test, p < 0.001). The overall RNA detection was higher for USUV, at 12.0% (303/2533; 95% CI: 10.8–13.3), than for WNV, at 4.3% (110/2533; 95% CI: 3.6–5.2), indicating dominance of USUV detections. Among migratory bird species (Fig 4A), USUV RNA prevalence was 11.0% (163/1484; 95% CI: 9.5–12.7), whereas WNV RNA prevalence was 3.4% (50/1484; 95% CI: 2.6–4.4). Similarly, among sedentary bird species (Fig 4B), USUV was detected in 13.3% of birds (140/1049; 95% CI: 11.4–15.5), compared with 5.7% for WNV (60/1049; 95% CI: 4.5–7.3). Across both ecological groups, detection frequencies differed significantly between USUV and WNV (McNemar’s test, p < 0.001). Given the higher prevalence estimates for USUV compared with WNV, these results indicate a predominance of USUV detections in both sedentary and migratory birds. At the species level, relatively high prevalence values were observed in several sedentary species such as great tit (*Parus major*) (USUV: 17.6%, 31/176; WNV: 5.1%, 9/176) and house sparrow (*Passer domesticus*) (USUV: 21.5%, 32/149; WNV: 13.4%, 20/149). Among migratory species, detections were largely driven by highly sampled species such as barn swallow (*Hirundo rustica*) (USUV: 12.4%, 81/654; WNV: 2.4%, 16/654), while evidence of exposure to both viruses was observed in species such as European bee-eater (*Merops apiaster*) (USUV: 9.5%, 13/137; WNV: 8.8%, 12/137). Prevalence estimates varied widely within both groups, reflecting heterogeneous exposure patterns as well as the influence of sample size, with higher values often observed in species with limited numbers of individuals. Overall, these results indicate that WNV/USUV RNA was detected in both ecological groups, with a higher detection rate in sedentary than in migratory species.

Across both years, co-infections with USUV and WNV remained uncommon, accounting for 2.0% of all sampled birds (51/2533; 95% CI: 1.5–2.6), and were detected in a limited set of host species, with marked heterogeneity in molecular prevalence across species. Co-infections were markedly more frequent in 2024, with 42 double-positive individuals (4.1%; 42/1032; 95% CI: 3.0–5.5), than in 2025, when only 9 cases were detected (0.6%; 9/1520; 95% CI: 0.3–1.1) (Fisher’s exact test, p < 0.001). When considering the cumulative dataset, co-infection prevalence ranged from less than 1% in highly sampled species, such as the barn swallow (0.8%, 5/654) to values exceeding 10% in species with smaller sample sizes, including the bearded reedling. Among common sedentary species, co-infections were observed in the house sparrow (8.1%, 12/149) and the great tit (4.0%, 7/176), whereas migratory species generally exhibited lower co-infection. Several other species showed sporadic co-infections at low levels (≤4%), reflecting limited but recurrent overlap of USUV and WNV circulation. Taken together, these results suggest that co-infections represent a rare epidemiological outcome, unevenly distributed among host species, and not restricted to a specific ecological guild or taxonomic group.

### Mixed-effects analysis of USUV and WNV detection

The distribution of individuals across the explanatory-variable categories is presented in S2 Table. Separate univariable and multivariable mixed-effects logistic regression models were fitted for USUV and WNV detection, with bird species included as a random effect (Table 1). Both final multivariable models retained the same explanatory variables: year, season, locality type and age. For USUV, the fixed effects accounted for 8.7% of the variance (marginal R^2^ = 0.087), while the full model explained 15.1% (conditional R^2^ = 0.151). The WNV model showed substantially higher explanatory power, with 49.8% of the variance explained by the fixed effects alone and 54.6% by the full model (marginal R^2^ = 0.498; conditional R^2^ = 0.546). Species-level clustering remained modest in both models, accounting for 7.0% of the variance in USUV detection (ICC = 0.070) and 9.5% in WNV detection (ICC = 0.095).

**Table 1:** Univariable and multivariable mixed-effects logistic regression models for USUV and WNV detection in wild birds. Results are presented as odds ratios (OR), 95% confidence intervals (95% CI) and p-values. Values in bold indicate statistically significant associations at p < 0.05. Reference categories are indicated as “Ref”.

			Univariable			Multivariable		
Virus	Variable	Category	OR	95% CI	p-value	OR	95% CI	p-value
USUV	Migration pattern	Sedentary	Ref	—	—			
		Migratory	0.9	0.5–1.4	0.551			
	Order	Non-Passeriformes	Ref	—	—			
		Passeriformes	1.1	0.5–2.4	0.794			
	Year	2024	Ref	—	—	Ref	—	—
		2025	0.6	0.5–0.8	**<0.001**	0.7	0.5–0.9	**0.006**
	Season	Summer	Ref	—	—	Ref	—	—
		Autumn/Winter	0.4	0.2–0.6	**<0.001**	0.4	0.2–0.6	**<0.001**
		Spring	0.8	0.6–1.1	0.142	0.8	0.6–1.2	0.273
	Locality	Rural	Ref	—	—	Ref	—	—
		Urban/Peri-urban	0.4	0.2–0.6	**<0.001**	0.5	0.2–0.8	**0.010**
	Age	1A	Ref	—	—	Ref	—	—
		2A+	1.0	0.6–1.5	0.902	1.0	0.6–1.5	0.851
		PUL	2.1	1.3–3.5	**0.004**	2.0	1.2–3.4	**0.014**
		VOL	2.5	1.5–4.5	**0.001**	2.6	1.5–4.7	**<0.001**
	Sex	Female	Ref	—	—			
		Male	0.9	0.6–1.4	0.686			
WNV	Migration pattern	Sedentary	Ref	—	—			
		Migratory	0.9	0.4–1.9	0.784			
	Order	Non-Passeriformes	Ref	—	—			
		Passeriformes	0.4	0.2–1.0	0.061			
	Year	2024	Ref	—	—	Ref	—	—
		2025	0.2	0.2–0.4	**<0.001**	0.1	0.1–0.2	**<0.001**
	Season	Summer	Ref	—	—	Ref	—	—
		Autumn/Winter	0.1	0.0–0.5	**0.004**	0.1	0.0–0.5	**0.003**
		Spring	5.1	3.2–8.1	**<0.001**	9.1	5.2–16.2	**<0.001**
	Locality	Rural	Ref	—	—	Ref	—	—
		Urban/Peri-urban	0.2	0.1–0.7	**0.009**	0.3	0.1–1.0	**0.049**
	Age	1A	Ref	—	—	Ref	—	—
		2A+	1.7	0.9–3.2	0.138	1.5	0.7–2.9	0.282
		PUL	8.9	4.7–17.0	**<0.001**	2.8	1.4–5.7	**0.005**
		VOL	0.6	0.2–2.0	0.441	2.5	0.7–8.2	0.145
	Sex	Female	Ref	—	—			
		Male	1.4	0.8–2.6	0.256			

Although virus detections were observed in both migratory and sedentary birds, migratory status was not significantly associated with USUV (OR = 0.9, 95% CI: 0.5–1.4) or WNV (OR = 0.9, 95% CI: 0.4–1.9) detection in the univariable mixed-effects models and was therefore not retained in the final multivariable models.

Regarding individual bird characteristics, sex was not significantly associated with WNV (OR = 1.4, 95% CI: 0.8–2.6) or USUV (OR = 0.9, 95% CI: 0.6–1.4) positivity. By contrast, age was retained in both final models, with virus positivity differing significantly among age classes for WNV and USUV. For WNV, the highest positivity rate was observed in *pullus* (PUL; 17.6%; OR = 2.8, 95% CI: 1.4–5.7), corresponding to the nesting stage characterized by dependence and absence of flight capability, whereas all other age classes showed lower rates. For USUV, volant individuals (VOL; 22.8%; OR = 2.6, 95% CI: 1.5–4.7), corresponding to the fledgling stage marked by flight ability and increasing autonomy, and PUL (18.3%; OR = 2.0, 95% CI: 1.2–3.4) exhibited the highest proportions of positive individuals, while older age classes consistently displayed lower positivity. Age was significantly associated with virus positivity, with juvenile stages, particularly *pullus* and volant individuals, showing higher positivity compared to older age classes. Most captured birds belonged to insectivorous species, either strictly insectivorous (64%) or insectivorous during the breeding season and granivorous in winter (30%). Because the distribution across dietary guilds was highly unbalanced, with very small numbers in several categories, feeding guild was not included in the mixed-effects models.

### Temporal Dynamics and Spatial Distribution of USUV and WNV

The distribution of USUV and WNV detections revealed significant inter-annual differences, with lower detection probabilities in 2025 than in 2024 for both viruses according to the mixed-effects models (USUV: OR = 0.7, 95% CI: 0.5–0.9; WNV: OR = 0.1, 95% CI: 0.1–0.2). In 2024, the overall prevalence reached 14.0% for USUV (144/1032; 95% CI: 12.0–16.2) and 7.7% for WNV (79/1032; 95% CI: 6.2–9.4). In 2025, lower prevalence levels were observed, with 10.5% for USUV (159/1520; 95% CI: 9.0–12.1) and 2.0% for WNV (31/1520; 95% CI: 1.4–2.9). This shift appeared to be associated with a reduction in WNV detections and a more moderate decrease in USUV prevalence, while USUV remained the most frequently detected virus in both years.

**Fig 5:**
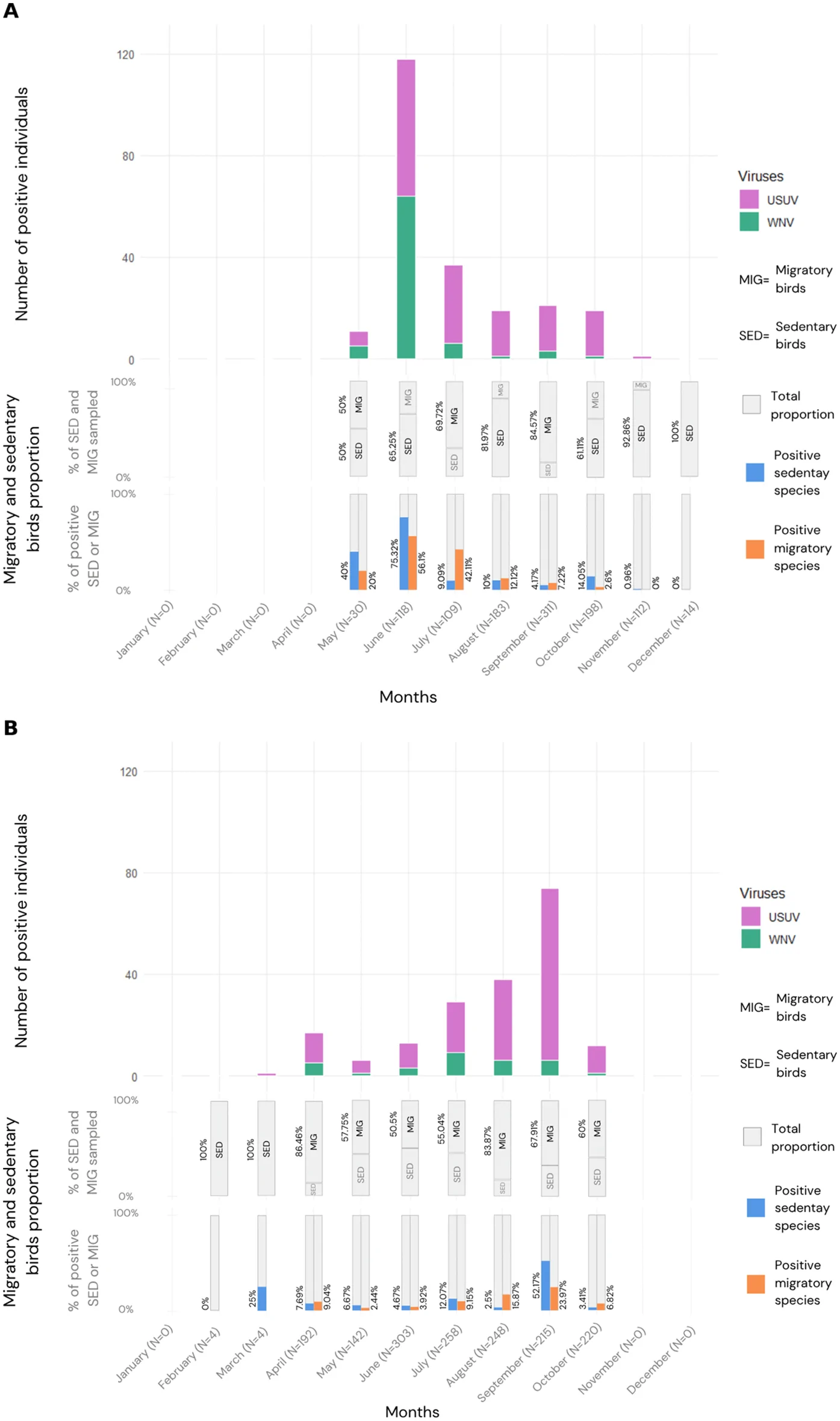
Monthly distribution of WNV- and USUV-positive birds in 2024 (A) and 2025 (B). Top panels show the number of individuals testing positive for WNV (green) and USUV (purple) by month; the total number of individuals sampled each month is indicated in parentheses on the x-axis. Bottom panels show, for each month, the proportion of sampled individuals that were migratory (in blue) versus sedentary (in orange), and the proportion of positive detections attributable to each migratory status among the individuals sampled that month.

The monthly distribution of USUV and WNV detections revealed distinct patterns. In 2024 (Fig 5A; Table 2A), viral activity was concentrated between May and October, with a pronounced synchronous peak in June (WNV: 64 positives, 54.2%; USUV: 54 positives, 45.8%), representing the highest level of detection for both viruses. Initial detections occurred in May, corresponding to the first month of sampling in 2024 (WNV: 5, 16.7%; USUV: 6, 20.0%). In July, WNV detections declined markedly (6 positives, 5.5%), while USUV remained relatively elevated (31 positives, 28.4%). From August to October, USUV persisted at moderate levels (18 positives per month; 5.8–9.8%), whereas WNV circulation remained sporadic (≤3 positives per month, ≤ 1.0%). No viral detections were recorded in November–December for WNV, and USUV dropped to minimal levels (1 case in November). In 2025 (Fig 5B; Table 2B), viral activity followed a different temporal pattern characterized by an extended transmission window. USUV detections began as early as March (1 positive, 25.0%, based on small sample size) and increased progressively through summer, peaking in September (68 positives, 31.6%), before declining in October (11 positives, 5.0%). In contrast, WNV circulation remained limited throughout the year, with low-level detections from April to October and a modest peak in July (9 positives, 3.5%). Overall, 2024 was characterized by an early-summer synchronous amplification of both viruses, whereas 2025 displayed a prolonged USUV transmission season with a delayed late-summer/early-autumn peak and persistently low WNV activity. Sampling intensity varied across months, with lower numbers of samples tested in early and late periods, resulting in wider confidence intervals for some estimates. Consistent with these descriptive patterns, season was retained in both final mixed-effects models. For analyses, month of sampling was grouped into seasons as follows: winter, January-March; spring, April-June; summer, July-September; and autumn, October-December. Compared with summer, autumn/winter was associated with lower detection probabilities for both USUV (OR = 0.4, 95% CI: 0.2–0.6) and WNV (OR = 0.1, 95% CI: 0.0–0.5), whereas spring was associated with a markedly higher probability of WNV detection but not with a significant difference in USUV detection.

**Table 2:** Monthly and locality-based prevalence of West Nile virus (WNV) and Usutu virus (USUV) in 2024 and 2025. Panel A shows the monthly and locality-based RNA prevalence of WNV and USUV in 2024, while Panel B presents the same data for 2025. For each month and locality, the number of positive samples (n), prevalence (%), and 95% confidence interval (95% CI) are reported for both viruses, along with the total number of samples tested.

	WNV positive (n)	RNA Prevalence %	95% CI	USUV positive (n)	RNA Prevalence %	95% CI	N Total
**A)**							
**Month of sampling in 2024**							
Mai	5	16.7%	7.3–33.6	6	20.0%	9.5–37.3	30
Juin	64	54.2%	45.3–63.0	54	45.8%	37.1–54.7	118
Juillet	6	5.5%	2.6–11.5	31	28.4%	20.8–37.5	109
Aout	1	0.6%	0.1–3.0	18	9.8%	6.3–15.0	183
Septembre	3	1.0%	0.3–2.8	18	5.8%	3.7–9.0	311
Octobre	1	0.5%	0.1–2.8	18	9.1%	5.8–13.9	198
Novembre	0	0.0%	0.0–3.3	1	0.9%	0.2–4.9	112
Décembre	0	0.0%	0.0–21.5	0	0.0%	0.0–21.5	14
**Locality**							
Aigues-Mortes	1	10.0%	1.8–40.4	0	0.0%	0.0–27.8	10
Aigues-Vives	34	8.0%	5.8–11.0	66	15.5%	12.4–19.3	425
Montarnaud	0	0.0%	0.0–22.8	1	7.7%	1.4–33.3	13
Rouet	0	0.0%	0.0–65.8	0	0.0%	0.0–65.8	2
Saint-Gilles	4	80.0%	37.6–96.4	1	20.0%	3.6–62.5	5
Saint-Laurent-D’Aigouze	22	4.2%	2.8–6.2	58	11.0%	8.6–14.0	527
Vauvert	18	36.0%	24.1–49.9	18	36.0%	24.1–49.9	50
**B)**							
**Month of sampling in 2025**							
Février	0	0.0%	0.0–49.0	0	0.0%	0.0–49.0	4
Mars	0	0.0%	0.0–49.0	1	25.0%	4.6–70.0	4
Avril	5	2.6%	1.1–6.0	12	6.3%	3.1–10.6	192
Mai	1	0.7%	0.1–3.9	5	3.5%	1.5–8.0	142
Juin	3	1.0%	0.3–2.9	10	3.3%	1.8–6.0	303
Juillet	9	3.5%	1.9–6.5	20	7.8%	5.1–11.7	258
Aout	6	2.4%	1.1–5.2	32	12.9%	9.3–17.7	248
Septembre	6	2.8%	1.3–6.0	68	31.6%	25.8–38.1	215
Octobre	1	0.5%	0.1–2.5	11	5.0%	2.8–8.7	220
**Locality**							
Agde	0	0.0%	0.0–3.2	3	2.6%	0.9–7.3	116
Aigues-Mortes	1	6.3%	1.1–28.3	2	12.5%	3.5–36.0	16
Aigues-Vives	3	1.1%	0.4–3.2	9	3.3%	1.8–6.2	272
Arles	0	0.0%	0.0–56.2	0	0.0%	0.0–56.2	3
Codognan	0	0.0%	0.0–79.4	0	0.0%	0.0–79.4	1
Le Cailar	0	0.0%	0.0–27.8	1	10.0%	1.8–40.4	10
Le Grau-du-Roi	0	0.0%	0.0–35.4	0	0.0%	0.0–35.4	7
Montarnaud	0	0.0%	0.0–49.0	1	25.0%	4.6–69.9	4
Montpellier	1	1.2%	0.2–6.5	6	7.2%	3.4–14.9	83
Murviel-lès-Montpellier	0	0.0%	0.0–65.8	0	0.0%	0.0–65.8	2
Rouet	1	1.1%	0.2–5.8	11	11.8%	6.7–20.0	93
Saint-Gilles	7	6.5%	3.2–12.8	5	4.6%	2.0–10.4	108
Saint-Laurent-d’Aigouze	7	1.9%	0.9–3.9	74	20.1%	16.3–24.5	368
Saintes-Maries-de-la-Mer	0	0.0%	0.0–39.0	0	0.0%	0.0–39.0	6
Vauvert	4	2.3%	0.9–5.7	28	15.9%	11.2–22.0	176
Villeneuve-lès-Maguelone	7	2.8%	1.3–5.6	19	7.5%	4.8–11.3	255

**Fig 6:**
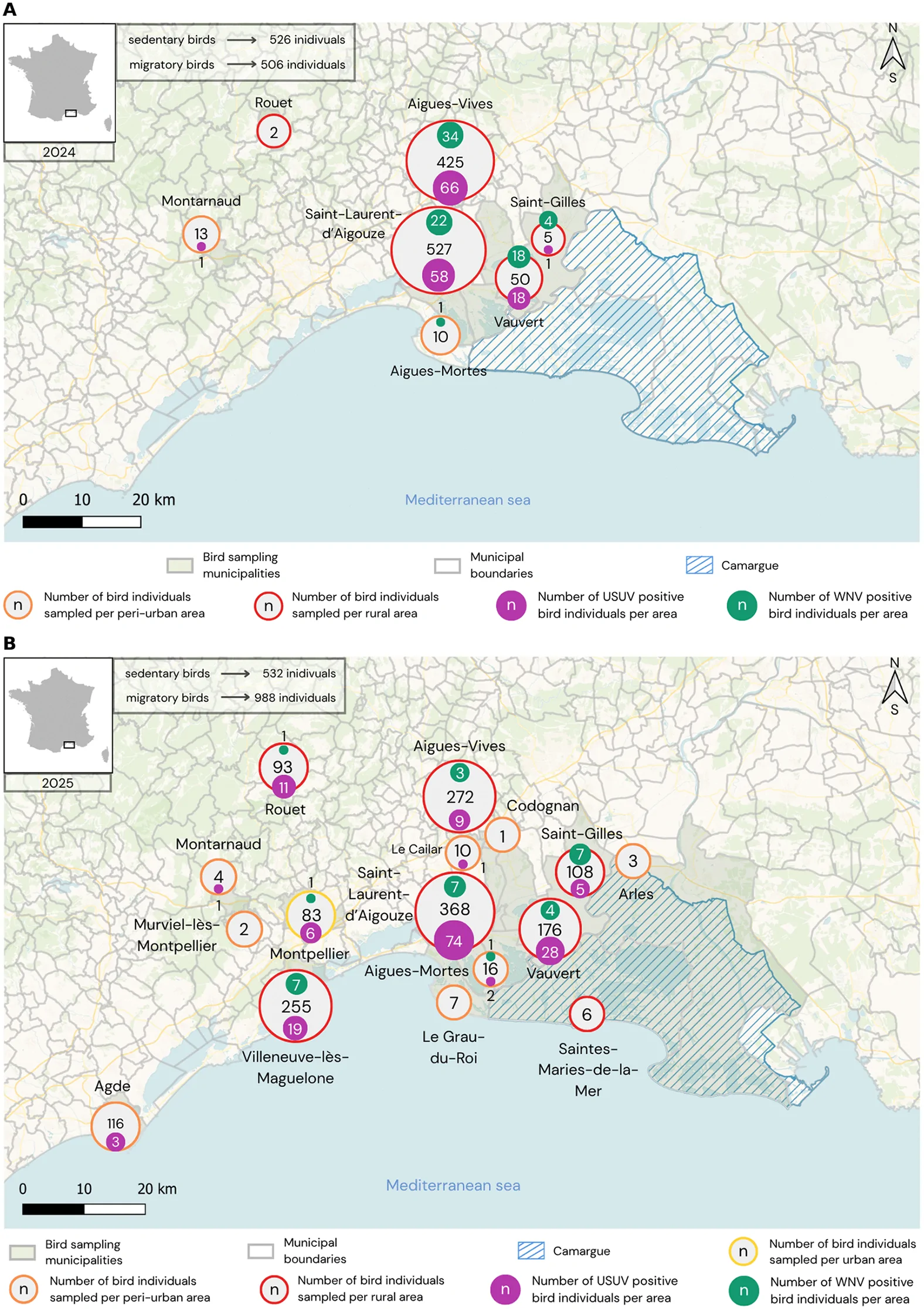
Spatial distribution of USUV and WNV infections in birds captured in 2024 (A) and 2025 (B). Each circle is centred on a sampling municipality; its size and inset number indicate the total number of individual birds captured there, and its outline colour indicates the locality type as defined in the Methods (yellow: urban; orange: peri-urban; red: rural). Smaller purple and green circles indicate, respectively, the number of USUV-positive and WNV-positive individuals identified at each site; their size is scaled to the number of positive individuals, so that positive counts can be interpreted relative to local sampling effort. Hatched areas delineate the Camargue wetland complex. A scale bar (0–20 km) and north arrow are provided. The inset map in the top-left corner situates the study area within France and summarises the total numbers of sedentary and migratory individuals sampled that year. Base map and overlay layers as described in Fig 1. Base map: CARTO Voyager No Labels (https://carto.com/basemaps/); map tiles CARTO, licensed CC BY 3.0 (https://creativecommons.org/licenses/by/3.0/); map data OpenStreetMap contributors, licensed under the Open Database License (ODbL) (https://www.openstreetmap.org/copyright; licence text: https://opendatacommons.org/licenses/odbl/1-0/index.html). Overlay layers: French municipal boundaries and Parcs Naturels Régionaux (PNR) boundaries, derived from OpenStreetMap and distributed via data.gouv.fr under ODbL. https://www.data.gouv.fr/datasets/parcs-naturels-regionaux-pnr-france-metropolitaine; https://www.data.gouv.fr/datasets/decoupage-administratif-communal-francais-issu-d-openstreetmap.

Spatially, viral circulation displayed a heterogeneous distribution across sampling sites, with differences observed between 2024 and 2025. In 2024 (Fig 6A; Table 2A), WNV detections were recorded across several localities, including rural areas (Saint-Gilles, Vauvert, Saint-Laurent-d’Aigouze,Aigues-Vives); however, site-specific proportions should be interpreted with caution due to limited sample sizes in some locations. In contrast, USUV detections were more widely distributed, with higher proportions observed in rural areas such as Vauvert (36.0%), Saint-Laurent-d’Aigouze (11.0%), and Aigues-Vives (15.5%), providing more robust site-level estimates. In 2025 (Fig 6B; Table 2B), USUV activity remained substantial across several rural sites, including Saint-Laurent-d’Aigouze (20.1%), Vauvert (15.9%), and Rouet (11.8%). In contrast, WNV prevalence remained low across most sites, with slightly higher values observed in Saint-Gilles (6.5%) and Villeneuve-lès-Maguelone (2.8%). Notably, a small number of USUV-positive birds (n = 6) and a single WNV-positive bird were also detected in 2025 within the city of Montpellier, the only urban site, indicating the presence of viral circulation in urban environments. Across both years combined, the highest proportion of positive cases was observed in rural areas (17.5%; 396/2268), followed by urban (8.4%; 7/83) and peri-urban areas (5.5%; 10/182). This spatial pattern was supported by the mixed-effects analyses, in which locality type was retained in both final models and detection probabilities were lower in urban/peri-urban areas than in rural areas for both USUV (OR = 0.5, 95% CI: 0.2–0.8) and WNV (OR = 0.3, 95% CI: 0.1–1.0).

**Fig 7:**
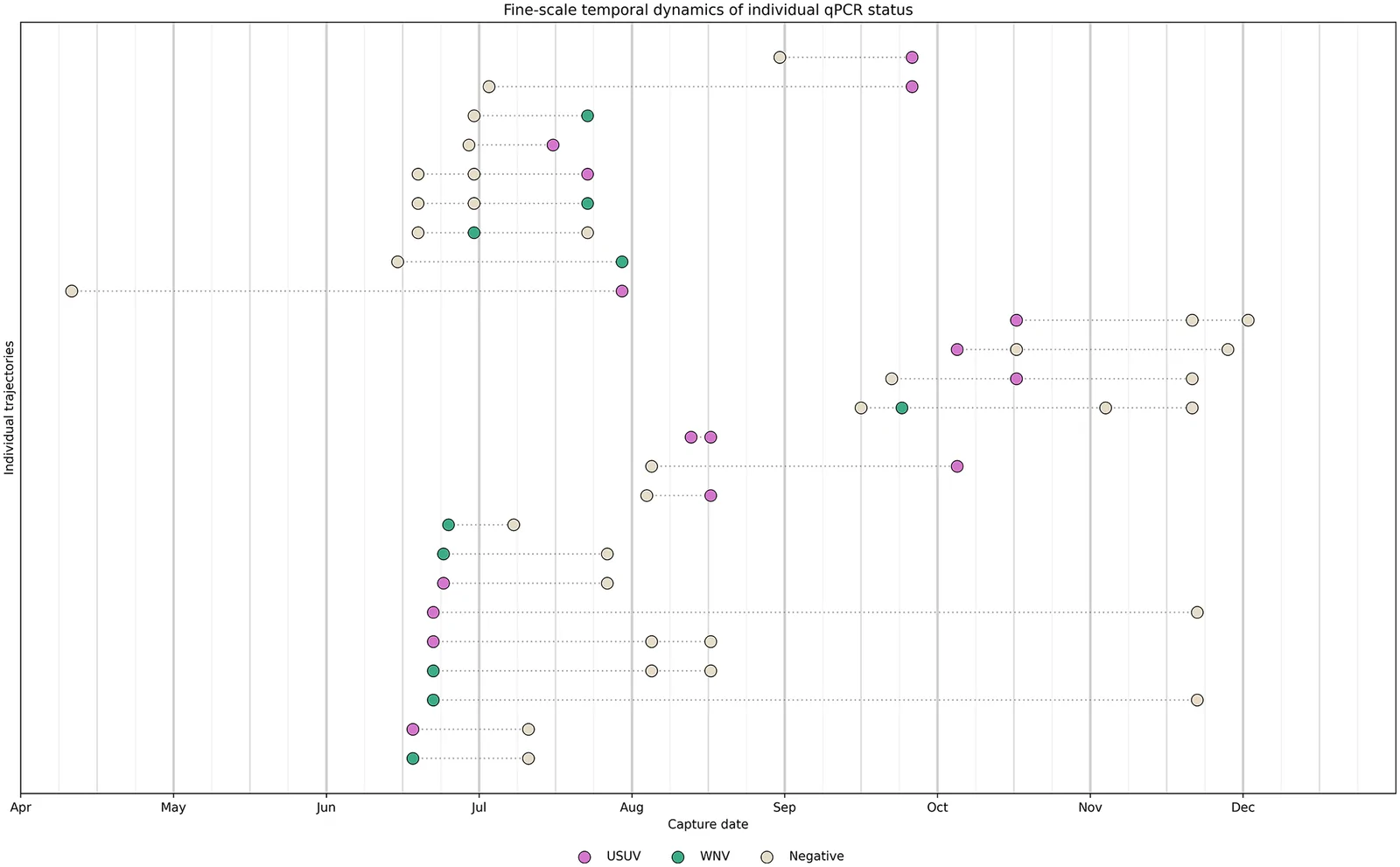
Fine-scale temporal dynamics of individual infection status in 2024 and 2025. Each point represents the positivity status observed at a given capture date for a single individual. Colors indicate viral status (USUV, WNV, or negative). Dashed lines connect successive captures of the same individual and indicate follow-up continuity only, without inferring infection status between capture events. Vertical background lines indicate calendar months (bold), mid-months (medium), and quarter-months (light). Each row along the y-axis corresponds to one sampled individual bird; the x-axis indicates the calendar date of each capture. Individuals contributing at least one positive detection and captured on more than one occasion, are shown.

A total of 93 birds were captured on at least two occasions within the same sampling year, allowing a longitudinal assessment of virological status at discrete capture dates (Fig 7). Although most individuals were tested negative at all capture dates, individual trajectories revealed temporal heterogeneity. Among birds that tested positive at least once, most corresponded either to individuals that were already positive at their first capture and subsequently became negative, for whom the onset of positivity could not be determined, or to individuals that were initially negative and became positive at a later capture, allowing in this case the identification of a bounded window of infection acquisition but without information on the timing of negativation. Three individuals exhibited a negative–positive–negative qPCR sequence. For these individuals, the maximum duration of infection was conservatively estimated as the interval between the last negative test preceding positivity and the first negative test following positivity. These intervals ranged from 24 to 41 days, providing upper bounds for the duration of infection given the discrete temporal resolution of the sampling. One individual exhibited two consecutive positive qPCR results for USUV across captures separated by four days. Additional sequential sampling would be necessary to refine the duration of detection according to species. No individual showed repeated positive detections over longer time intervals and no evidence of prolonged or long-term persistent qPCR positivity was observed at the temporal resolution of the sampling. This dataset offers limited power to detect persistence, however, as only three individuals contributed a bounded infection window (24–41 days) and a single individual contributed two consecutive positive results (4 days apart); within these constraints, no evidence of prolonged or long-term persistent RT-qPCR positivity was observed at the temporal resolution of the sampling.

### Partial sequencing and phylogenetic characterization of WNV and USUV

**Fig 8:**
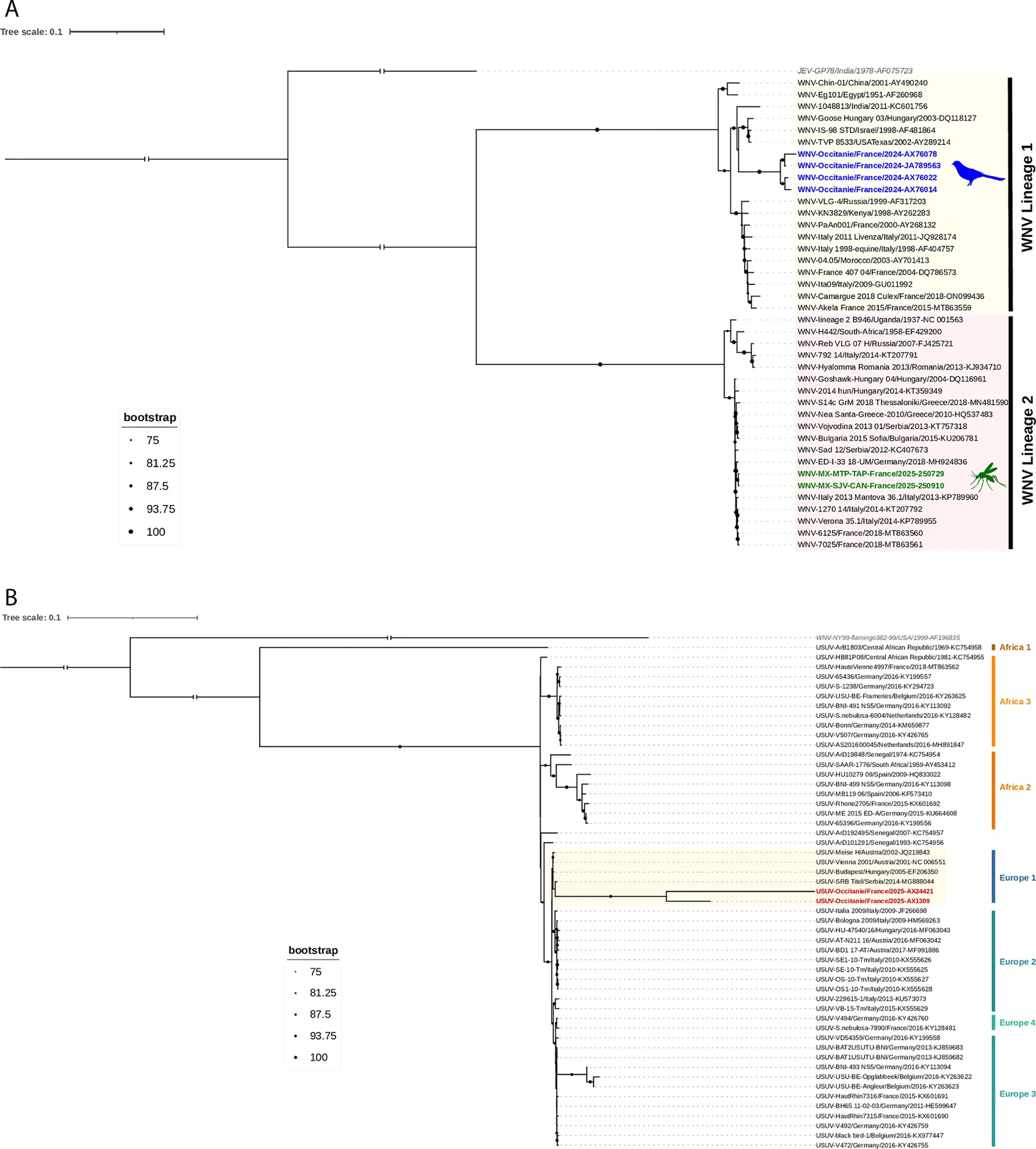
Phylogeny of representative sequences of WNV (A) and USUV (B). Trees were reconstructed in IQ-TREE 3.0.1 following alignment (MAFFT) and trimming (TrimAl) of the sequences generated in this study, alongside reference sequences retrieved from GenBank. Node support was assessed jointly using ultrafast bootstrap approximation (UFBoot2, 1,000 replicates) and the Shimodaira-Hasegawa-like approximate likelihood ratio test (SH-aLRT, 1,000 replicates); bootstrap values are displayed on the trees below, and SH-aLRT values for the same nodes are provided in the deposited tree files (see Data Availability). **(A)** Tree was rooted with a sequence of Japanese encephalitis virus (JEV) in grey. Sequences generated are shown in bold green for samples from mosquitoes and in bold blue for avian samples. Grey squares on branch indicate bootstrap support ≥75%. Scale bar represents nucleotide substitutions per site. (B) Phylogeny of representative sequences of Usutu Virus (USUV). Tree was rooted with a sequence of West Nile Virus (WNV) in grey. Sequences generated for this study are shown in bold red. Black points on branch indicate bootstrap support ≥75%. Scale bar represents nucleotide substitutions per site.

We were unable to obtain complete genome sequences from our samples due to the limited amount of material available from swabs or droppings. Nevertheless, several partial sequences were successfully generated, confirming sample positivity. Depending on amplicon length, a subset of these sequences was suitable for phylogenetic analysis and included in the trees shown in Fig 8. Sequence analysis of avian samples indicated the circulation of WNV lineage 1 in 2024, from which sequences were successfully obtained. In contrast, mosquito analyses conducted in 2025 revealed the presence of lineage 2 in our study area [44]. For USUV, phylogenetic analysis of the sequences generated in this study indicated that they clustered within the Europe 1 lineage, which has historically circulated mainly in southern and eastern Europe (Italy, Austria, Hungary, and Serbia). However, this lineage assignment is based on a limited number of partial sequences and should therefore be interpreted with caution.

### Birds as Sentinels of WNV and USUV Circulation: Seasonal Dynamics in 2024–2025

**Fig 9:**
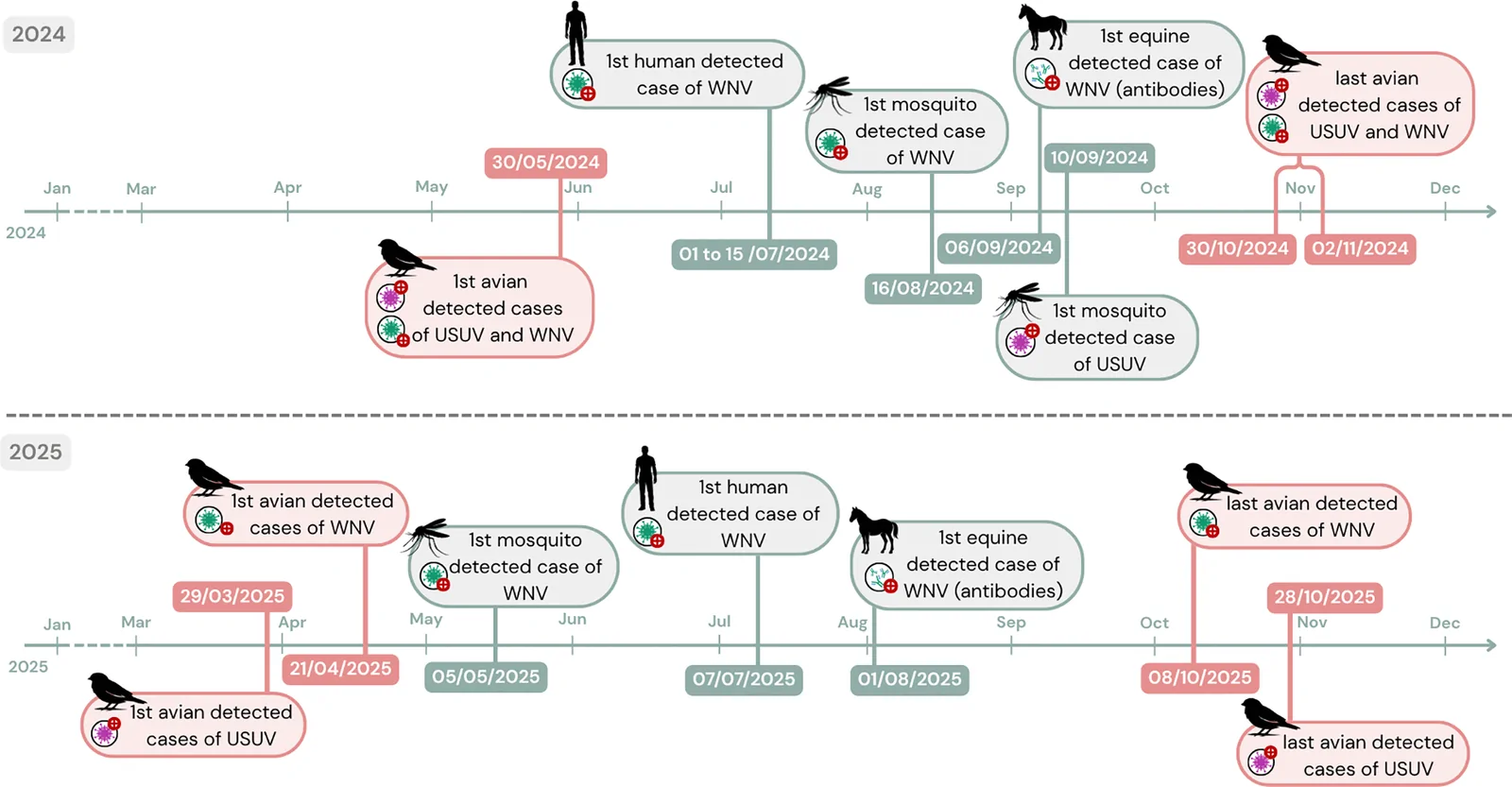
Temporal dynamics of WNV and USUV circulation across multiple surveillance matrices in 2024 and 2025 in Occitanie. Timeline showing first and last detections of WNV (green) and USUV (purple) in birds (orange), and only first detections of WNV and USUV in mosquitoes, equines, and humans (blue). Horizontal bars indicate the period between the first and last positive detection of each virus in a given sample matrix during each year. All bird data were generated in this study, whereas data on human cases were obtained from Santé Publique France, equine data from RESPE, and mosquito data from [45]. Silhouette illustrations generated using OpenAI, GPT-5.5, image generation and assembled by the authors (https://openai.com/policies/terms-of-use/).

In both years, avian sampling provided detection of WNV and USUV several weeks before virus detection in mosquito pools and before the occurrence of equine and human cases (Fig 9). In 2024, initial avian detections occurred in late May, preceding the first human WNV cases (early July), mosquito positives (early August) and equine cases (early September), with viral detections in birds extending into late October–early November. The 2025 season showed earlier onset with USUV-positive birds detected in late March, and WNV in avian samples and mosquito samples in late April, followed by the first WNV human case early July and WNV equine case in early August.

## Discussion

Our two-year avian molecular surveillance in the Camargue wetlands and urban and peri-urban areas of Montpellier (Occitanie, France) revealed sustained circulation of USUV and WNV. USUV detections were significantly more frequent than WNV detections, with overall RNA prevalences of 12.0% and 4.3%, respectively, and this predominance was observed in both sedentary and migratory bird species. Passeriformes represented the majority of viral detections in absolute numbers, largely because they constituted most of the sampled individuals (90.3%; 2288/2533). The order-level comparison between Passeriformes and the pooled remaining orders, a small and taxonomically heterogeneous group, should be interpreted with corresponding caution given this sampling imbalance. Within Passeriformes, several species contributed substantially to viral detections, particularly the great tit and house sparrow among sedentary species, and the barn swallow among migratory species. These species were also among the most heavily sampled, and their prominence in raw detection counts partly reflects sampling intensity rather than necessarily indicating higher biological susceptibility. This species-level pattern is consistent with European studies identifying several passerine species as primary reservoirs for WNV and USUV [46–48]. Their abundance, synanthropic behavior, and ecological overlap with ornithophilic *Culex pipiens* may favor frequent mosquito–host contact, as further supported by blood-meal analyses in the Camargue showing substantial feeding on passerines (≈42% of meals vs. ≈ 18% on waterbirds) [49,50]. Depending on the species, communal roosting, ground foraging and close association with human-modified environments may further increase exposure to vectors and help explain their frequent involvement in local virus circulation [49,51–53]. Experimental data support this interpretation, indicating that some passerines, such as the house sparrow, can develop viremia levels sufficient to sustain onward viral transmission [54]. Although detections outside Passeriformes were limited, several non-passeriform species showed noteworthy patterns. European rollers displayed a relatively high WNV detection rate (29.4%), while European bee-eaters exhibited circulation of both USUV and WNV across a relatively large sample size. These findings suggest that viral circulation was not restricted to passerine hosts and broaden the range of avian hosts documented in this local surveillance context, although such signals should be interpreted with caution given the limited sample sizes for several taxa.

The two consecutive years appeared to show a contrast in virus distribution patterns. In 2024, viral detections were distributed across multiple species and sites, with positives identified in 26 of 54 species across 6 of 7 monitored sites*.* In 2024 a pronounced peak occurred in June, temporally aligned with 12 autochthonous human WNV cases reported in Occitanie and 89 confirmed equine cases during the 2024 transmission season [15,55], recalling the 2015 Camargue epizootic [56]. In 2025, fewer positive birds were detected overall, and the barn swallow accounted for the largest number of USUV and WNV RNA detections. In Occitanie, 8 human cases and 11 equine cases of West Nile virus infection were reported that year, also fewer than in 2024 [57,58]. WNV circulation was mainly observed during spring and summer, whereas USUV was also detected during these seasons but extended into autumn, indicating a broader seasonal distribution. This pattern is consistent with recent European-wide syntheses, which increasingly report more widespread and frequent USUV activity than WNV, particularly in central and western Europe and in years with mild autumns [28,46,47,59,60]. Mechanistically, vector competence data indicate that local *Culex pipiens* populations can be highly susceptible to USUV, sometimes across broader temperature windows, enabling extended transmission into late autumn under temperate conditions [61,62]. Spatially, viral circulation was heterogeneous and significantly associated with locality type, with higher detection probabilities observed in rural environments compared to peri-urban areas, while urban sites showed no significant increase in risk despite evidence of low-level circulation. This pattern is consistent with the distribution of suitable mosquito habitats and host communities, with rural and wetland-associated environments providing favorable conditions for sustained transmission. Site-level observations further support this interpretation, with recurrent detections in several rural localities across both years, whereas urban circulation remained limited. Such interannual and spatio-temporal variability may plausibly be influenced by environmental conditions and vector dynamics: warmer and prolonged autumns can extend mosquito activity, allowing delayed amplification in birds and, subsequently, in humans [63–65]. Post-epidemic herd immunity may also have contributed, as the intense viral circulation observed in 2024 could have partially reduced susceptibility in 2025, thereby limiting multi-species amplification.

Our data are consistent with the possibility that migratory movements contribute to virus introduction while resident species help sustain local amplification, although this pattern cannot be directly demonstrated by cross-sectional RT-qPCR detection alone and should be regarded as a hypothesis rather than a demonstrated conclusion. Migratory movements likely facilitate the introduction of the virus, whereas resident species appear to sustain local amplification. In 2024 the earliest positive detections were observed in European bee-eaters (Merops apiaster) in May, shortly after their arrival in the study area (late April–early May), suggesting that infected migratory birds may enter the region already viremic and contribute to seeding local transmission. This chronology is consistent with previous studies showing that infected migratory birds can transport arboviruses over long distances along flyways [46,47,61]. However, a key unresolved question is whether migratory birds arrive already infected or acquire infection locally at stopover or breeding sites. Both pathways are plausible, given (i) phylogeographic and epidemiological evidence for long-distance viral transport and (ii) rapid local exposure where ornithophilic *Culex* overlap with dense bird aggregations [47]. Disentangling these pathways will require effort-adjusted analysis (e.g., occupancy or hierarchical models), longitudinal ringing/recapture, and targeted sequencing along migration routes to assign infection timing and origin [47].

In contrast to studies reporting demographic invariance in orthoflavivirus infection patterns [66], our results indicate that infection risk is structured by host age, as supported by multivariable analyses. For USUV, both juvenile stages (PUL and VOL) showed significantly higher detection probabilities compared to adults, whereas for WNV, this effect was primarily observed in PUL individuals, with no significant increase detected in VOL birds. These findings highlight the importance of individual-level demographic factors, particularly immunologically naïve cohorts, in shaping transmission dynamics within avian communities. Seasonal pulses of susceptible juveniles may therefore contribute disproportionately to viral amplification. In contrast, sex and dietary guild were not retained in the final multivariable model, suggesting that they do not constitute primary drivers of viral detection when considered alongside other factors. Given the limited subset of individuals for which sex was available, only partial patterns could be assessed. Within this subset, WNV detection appeared higher in males, whereas no clear difference was observed for USUV. These observations should be interpreted cautiously and may reflect sampling constraints rather than a consistent biological effect, although they could also be influenced by sex-specific behavioral patterns, habitat use, or physiological mechanisms affecting susceptibility.

We documented overlapping transmission of WNV and USUV across the season, with 51 co-infections detected among the 2,533 unique individuals sampled over the two years (2.0%). The relative rarity of co-infections, despite their occurrence across multiple host species, is consistent with ecological niche overlaps without extensive co-transmission. Co-infections were most frequently observed in species contributing the largest number of detections, particularly house sparrows and great tits, but were also identified in several other species, including barn swallows, European bee-eaters, and bearded reedlings. Interestingly, some species exhibited relatively high prevalence for both viruses but no observed co-infections (e.g., *Upupa epops*), suggesting that co-infection is not solely driven by exposure but may also depend on within-host processes, the timing of infection, or limited sample size, potentially reflecting asynchronous exposure, transient infection windows, or biological interactions limiting co-occurrence at the individual level. These observations indicate broader host overlap rather than confinement to a single ecological group and suggest spatial and temporal niche overlap where *Culex* populations are exposed to both viruses [15,47]. Although *in vitro* data indicate that USUV may competitively constrain WNV, the population‑level implications of co‑infections remain uncertain. Co-circulation of WNV and USUV raises questions regarding lineage-level interactions at the vector–host interface, including competition, facilitation, or cross-immunity. Experimental evidence supports possible antagonistic interactions, as USUV has been shown *in vitro* to inhibit WNV replication via interferon-mediated pathways, potentially contributing to reduced WNV amplification in areas of intense USUV circulation [67]. However, field evidence remains limited and sometimes contradictory, and these interactions are likely modulated by ecological drivers such as host availability, temperature regimes, and vector phenology [46,47]. From an analytical perspective, the explicit characterization of co-infection distribution in our dataset provides initial insight into shared transmission dynamics. However, extending this approach through dedicated modeling of co-infection patterns, in a framework comparable to that used for single-virus infection, could further disentangle shared and virus-specific drivers of transmission and better quantify the ecological and epidemiological conditions under which co-infections occur. Methodologically, these findings also support the use of multiplex PCR and lineage-aware confirmation (sequencing) to avoid misclassification, detect shifts in viral dominance across seasons, and integrate entomological and avian data within a One Health surveillance framework [15,47,59,60].

Phylogeographic analyses indicate that multiple USUV lineages have entered Europe [33,47,68]. In parallel, WNV lineage 2 has progressively replaced lineage 1 across much of Europe and underpins most recent large human and equine outbreaks [44,47]. Obtaining robust phylogenetic signal from cloacal swabs or droppings remains challenging, as these samples often yield partial genomes or lower viral loads compared to tissue samples, which can limit the resolution of lineage assignments. 2024–2025 data show exclusive circulation of WNV lineage 1 in 2024 and a shift to lineage 2 in 2025, contrasting with the historical dominance of lineage 1 in this region and reinforcing evidence for the stable establishment of lineage 2 in southern France, consistent with broader European trends observed since 2010 [15,69,70]. For USUV, we identified a strain related to the Europe 1 lineage, although low nodal support at this position warrants cautious interpretation, this lineage has historically been reported circulating in southern and eastern Europe since its emergence in Austria (2001) and Italy (1996) [3,71].

Despite high molecular detection across both years, we observed no conspicuous avian die-offs in the study area. This silent circulation is consistent with several, non-exclusive mechanisms: (i) the infected pool was dominated by tolerant hosts, notably passerines, in which infections are frequently subclinical or mildly symptomatic; (ii) lineage–host interactions likely modulate virulence producing context-dependent outcomes; and (iii) under-ascertainment of carcasses in open wetlands and agricultural mosaics, where detection probability is low and carcasses are rapidly removed by scavengers. This pattern contrasts with documented USUV-associated mortality in blackbirds (*Turdus merula*) and corvids during epizootic years, including severe lesions and high case fatality in experimental infections (with AF3/E3 lineages) [72]. Field reports of large blackbird die-offs in several outbreaks highlight strong interspecific differences in pathogenicity and suggest that passive surveillance systems (on dead birds) are biased toward susceptible, conspicuous taxa (corvids, raptors), potentially underestimating burden in passerines where disease is rarely lethal. Consequently, absence of mass mortality should not be interpreted as low transmission risk: subclinical infections in abundant passerines can sustain high viral circulation and raise spillover potential under favorable vector conditions [46].

Avian detection consistently preceded detections in mosquitoes, equines, and humans. These findings align with decades of evidence from Europe and North America showing that bird-based surveillance, whether through dead bird reporting, sentinel corvids, or targeted sampling offers superior timeliness compared to clinical case detection in incidental hosts [47,73,74]. Whether the magnitude of avian circulation additionally predicts the scale of subsequent human or equine caseload is a separate question that this two-year dataset cannot address, and detected avian RNA prevalence and regional human/equine case counts should not be assumed to scale together without further years of concurrent data. In an endemic region where the virus is already established locally, the temporal precedence of avian detection reflects the reservoir role of birds rather than a demonstrated forecasting capacity. Active avian surveillance is therefore best regarded as a complementary early-detection tool rather than a validated early-warning.

Several limitations should be acknowledged. Molecular detection, as opposed to serology, primarily captures active infections and may therefore underestimate past exposure and cumulative infection risk. In addition, uneven sampling effort across species, sites, and seasons may influence detection probabilities and contribute to apparent heterogeneity, particularly for species with small sample sizes. Sampling was designed to support active surveillance of viral circulation across the avian community as a whole, rather than to power comparisons between every species, site, or season; overall sample sizes are therefore substantial but unevenly distributed. This is especially relevant when interpreting the absence of detection or co-infection in certain taxa and calls for caution when interpreting results for the smallest or most unevenly sampled subgroups, which should be regarded as exploratory and descriptive rather than as robust estimates of effect. A further limitation is that spatial aggregations such as communal roosts may locally inflate infection rates and obscure broader-scale transmission patterns. In addition, although species-level effects were partially accounted for, unmeasured ecological variables (e.g., microhabitat use, vector density, or fine-scale host behavior) may also contribute to the observed variability. While co-infections were explicitly described, their drivers were not formally modeled, limiting inference on the mechanisms underlying their occurrence. Another consideration is that the binary classification of birds as migratory or sedentary does not capture the continuum of migratory strategies (e.g., partial migrants), which may introduce some classification uncertainty.

## Conclusion

Our results demonstrate widespread detections of WNV and USUV in several avian species in southern France. Unlike passive surveillance, which is often biased toward conspicuous mortality events or clinical cases, active sampling captures a broader range of host species, including those experiencing silent or subclinical infections, thereby providing a more complete picture of viral circulation. These findings support the continued value of active, non-invasive avian surveillance as a complement to mosquito, equine, and human monitoring within a One Health framework. However, the extent to which such surveillance can contribute to outbreak anticipation or guide operational public health responses remains to be evaluated through future studies using standardized sampling efforts and longer time series.

## Supporting information

S1 Table Summary of species traits and prevalence. The table presents species-level information including taxonomic identity, prevalence values, ecological traits, and associated classifications. For each species, prevalence represents the proportion of positive observations relative to the total number of individuals sampled, expressed as n/N with 95% confidence intervals (95% CI). USUV and WNV prevalences correspond to single infections only, whereas co-infection refers exclusively to simultaneous detection of both viruses in the same individual. Migration status is coded as follows: R = Resident; P = Partial migrant; S = Short-distance migrant; L = Long-distance migrant; W = Wintering; B = Breeding. SED/MIG 2024/2025 indicates the study-level classification of species as sedentary (SED) or migratory (MIG), and the year(s) of sampling (2024, 2025, or both). Diet category is coded as follows: INS = Insectivorous; INV = Invertivorous; OMN = Omnivorous; GRA = Granivorous; FRU = Frugivorous; CAR = Carnivorous; PIS = Piscivorous; m = migration; b = breeding; w = winter.
(DOCX)

S2 Table Distribution of sampled birds across the categories considered in the analyses. The table presents the number of individuals in each category according to year, season, locality type, age class, sex, migratory status, taxonomic group, and feeding guild. Sex was undetermined for 1488 individuals, and age information was missing for three individuals for both WNV and USUV datasets.
(DOCX)
