# Supplementary material for: Widespread circulation of West Nile and Usutu viruses in sedentary and migratory avifauna: A two-year study (2024–2025) of active surveillance in South of France
Source: PLoS Negl Trop Dis. 2026 Sep 3;20(9):e0014397. doi: 10.1371/journal.pntd.0014397 (PMC13568517; doi:10.1371/journal.pntd.0014397)
Supplement: Supplementary file 1 [file pntd.0014397.s001.docx]

| **Order** | **Family** | **Species** | **USUV prevalence**  **%**  **(n/N; 95% CI)** | **WNV prevalence**  **%**  **(n/N; 95% CI)** | **Co-infection**  **%**  **(n/N; 95% CI)** | **Migration pattern** | **SED/MIG**  **2024/2025** | **Feeding** |
| --- | --- | --- | --- | --- | --- | --- | --- | --- |
| Bucerotiformes | Upupidae | *Upupa*  *epops* | 33.3%  (1/3; 6.1–79.2) | 33.3%  (1/3; 6.1–79.2) | 0.0%  (0/3; 0.0–56.2) | P+W | MIG  2024 | INS |
| Caprimulgiformes | Caprimulgidae | *Caprimulgus*  *europaeus* | 33.3%  (1/3; 6.1–79.2) | 0.0%  (0/3; 0.0–56.2) | 0.0%  (0/3; 0.0–56.2) | L | MIG  2025 | INS |
| Charadriiformes | Larus | *Larus*  *michahellis* | 0.0%  (0/1; 0.0–79.3) | 0.0%  (0/1; 0.0–79.3) | 0.0%  (0/1; 0.0–79.3) | R | SED  2024 | OMN |
|  | Scolopacidae | *Actitis*  *hypoleucos* | 0.0%  (0/1; 0.0–79.3) | 0.0%  (0/1; 0.0–79.3) | 0.0%  (0/1; 0.0–79.3) | R+P | SED  2024 | INV |
|  |  | *Gallinago*  *gallinago* | 0.0%  (0/1; 0.0–79.3) | 0.0%  (0/1; 0.0–79.3) | 0.0%  (0/1; 0.0–79.3) | L+W | MIG  2025 | INV |
| Coraciiformes | Alcedinidae | *Alcedo*  *atthis* | 4.2%  (1/24; 0.7–20.2) | 0.0%  (0/24; 0.0–13.8) | 0.0%  (0/24; 0.0–13.8) | R+P | SED  2024/2025 | PIS |
|  | Coraciidae | *Coracias*  *garrulus* | 0.0%  (0/17; 0.0–18.4) | 23.5%  (4/17; 9.6–47.3) | 5.9%  (1/17; 1.0–27.0) | L+B | MIG  2024/2025 | INS |
|  | Meropidae | *Merops*  *apiaster* | 6.6%  (9/137; 3.5–12.0) | 5.8%  (8/137; 3.0–11.1) | 2.9%  (4/137; 1.1–7.3) | L+B | MIG  2024/2025 | INS |
| Passeriformes | Acrocephalidae | *Acrocephalus*  *arundinaceus* | 9.5%  (4/42; 3.8–22.1) | 0.0%  (0/42; 0.0–8.4) | 4.8%  (2/42; 1.3–15.8) | L+B | MIG  2024/2025 | INS |
|  |  | *Acrocephalus*  *melanopogon* | 16.7%  (12/72; 9.8–26.9) | 4.2%  (3/72; 1.4–11.5) | 4.2%  (3/72; 1.4–11.5) | R+P | SED  2024/2025 | INS |
|  |  | *Acrocephalus*  *paludicola* | 0.0%  (0/1; 0.0–79.3) | 0.0%  (0/1; 0.0–79.3) | 0.0%  (0/1; 0.0–79.3) | L | MIG  2025 | INS |
|  |  | *Acrocephalus*  *schoenobanus* | 10.0%  (2/20; 2.8–30.1) | 0.0%  (0/20; 0.0–16.1) | 0.0%  (0/20; 0.0–16.1) | L | MIG  2024/2025 | INS |
|  |  | *Acrocephalus*  *scirpaceus* | 11.7%  (26/223; 8.1–16.5) | 0.9%  (2/223; 0.2–3.2) | 1.3%  (3/223; 0.5–3.9) | S+B | MIG  2024/2025 | INS |
|  | Certhiidae | *Certhia*  *brachydactyla* | 0.0%  (0/7; 0.0–35.4) | 0.0%  (0/7; 0.0–35.4) | 0.0%  (0/7; 0.0–35.4) | R | SED  2025 | INS |
|  | Cettiidae | *Cettia*  *cetti* | 27.9%  (12/43; 16.7–42.7) | 2.3%  (1/43; 0.4–12.1) | 0.0%  (0/43; 0.0–8.2) | R | SED  2024/2025 | INS |
|  | Cisticolidae | *Cisticola*  *juncidis* | 0.0%  (0/2; 0.0–65.8) | 0.0%  (0/2; 0.0–65.8) | 0.0%  (0/2; 0.0–65.8) | R | SED  2025 | INS |
|  | Corvus | *Corvus*  *corone* | 0.0%  (0/1; 0.0–79.3) | 0.0%  (0/1; 0.0–79.3) | 0.0%  (0/1; 0.0–79.3) | R | SED  2024 | OMN |
|  | Emberizidae | *Emberiza*  *cirlus* | 0.0%  (0/2; 0.0–65.8) | 0.0%  (0/2; 0.0–65.8) | 0.0%  (0/2; 0.0–65.8) | R | SED  2025 | INS(b) + GRA(w) |
|  |  | *Emberiza*  *schoeniclus*  *schoeniclus* | 1.3%  (1/76; 0.2–7.1) | 1.3%  (1/76; 0.2–7.1) | 0.0%  (0/76; 0.0–4.8) | S+W | MIG  2024/2025 | INS(b) + GRA(w) |
|  |  | *Emberiza*  *schoeniclus*  *witherbyi* | 8.7%  (2/23; 2.4–26.8) | 0.0%  (0/23; 0.0–14.3) | 0.0%  (0/23; 0.0–14.3) | R | SED  2024/2025 | INS(b) + GRA(w) |
|  |  | *Milaria*  *calandra* | 18.8%  (3/16; 6.6–43.0) | 0.0%  (0/16; 0.0–19.4) | 0.0%  (0/16; 0.0–19.4) | R | SED  2024/2025 | INS(b) + GRA(w) |
|  | Fringillidae | *Carduelis*  *carduelis* | 5.7%  (4/70; 2.2–13.8) | 0.0%  (0/70; 0.0–5.2) | 4.3%  (3/70; 1.5–11.9) | R+P | SED  2024/2025 | INS(b) + GRA(w) |
|  |  | *Carduelis*  *chloris* | 7.4%  (2/27; 2.1–23.4) | 0.0%  (0/27; 0.0–12.5) | 0.0%  (0/27; 0.0–12.5) | R+P | SED  2024/2025 | INS(b) + GRA(w) |
|  |  | *Coccothraustes*  *coccothraustes* | 0.0%  (0/1; 0.0–79.3) | 0.0%  (0/1; 0.0–79.3) | 0.0%  (0/1; 0.0–79.3) | R+P | SED  2025 | INS(b) + GRA(w) |
|  |  | *Fringilla*  *coelebs* | 0.0%  (0/5; 0.0–43.4) | 0.0%  (0/5; 0.0–43.4) | 0.0%  (0/5; 0.0–43.4) | R+P | MIG  2024/2025 | INS(b) + GRA(w) |
|  |  | *Serinus*  *serinus* | 6.2%  (3/48; 2.1–16.8) | 4.2%  (2/48; 1.2–14.0) | 0.0%  (0/48; 0.0–7.4) | R | SED  2024/2025 | INS(b) + GRA(w) |
|  | Hippolais | *Hippolais*  *polyglotta* | 0.0%  (0/10; 0.0–27.8) | 0.0%  (0/10; 0.0–27.8) | 0.0%  (0/10; 0.0–27.8) | L+B | MIG  2024/2025 | INS |
|  | Hirundinidae | *Hirundo*  *rustica* | 11.6%  (76/654; 9.4–14.3) | 1.7%  (11/654; 0.9–3.0) | 0.8%  (5/654; 0.3–1.8) | L+B | MIG  2024/2025 | INS |
|  |  | *Riparia*  *riparia* | 4.8%  (2/42; 1.3–15.8) | 0.0%  (0/42; 0.0–8.4) | 2.4%  (1/42; 0.4–12.3) | L | MIG  2024/2025 | INS |
|  | Laniidae | *Lanius*  *collurio* | 0.0%  (0/1; 0.0–79.3) | 0.0%  (0/1; 0.0–79.3) | 0.0%  (0/1; 0.0–79.3) | L | MIG  2024 | CAR |
|  |  | *Lanius*  *senator* | 0.0%  (0/2; 0.0–65.8) | 0.0%  (0/2; 0.0–65.8) | 0.0%  (0/2; 0.0–65.8) | L | MIG  2024/2025 | CAR |
|  | Locustellidae | *Locustella*  *naevia* | 0.0%  (0/6; 0.0–39.0) | 0.0%  (0/6; 0.0–39.0) | 0.0%  (0/6; 0.0–39.0) | L | MIG  2025 | INS |
|  | Motacillidae | *Motacilla*  *flava* | 6.2%  (4/64; 2.5–15.0) | 1.6%  (1/64; 0.3–8.3) | 1.6%  (1/64; 0.3–8.3) | L+B | MIG  2024/2025 | INS |
|  | Muscicapidae | *Erithacus*  *rubecula* | 14.3%  (3/21; 5.0–34.6) | 0.0%  (0/21; 0.0–15.5) | 0.0%  (0/21; 0.0–15.5) | R+P | SED  2024/2025 | INS(b) + GRA(w) |
|  |  | *Ficedula*  *hypoleuca* | 22.2%  (2/9; 6.3–54.7) | 0.0%  (0/9; 0.0–29.9) | 0.0%  (0/9; 0.0–29.9) | S | MIG  2024/2025 | INS |
|  |  | *Luscinia*  *megarhynchos* | 7.7%  (2/26; 2.1–24.1) | 3.8%  (1/26; 0.7–18.9) | 3.8%  (1/26; 0.7–18.9) | S+B | MIG  2024/2025 | INS |
|  |  | *Luscinia*  *svecica* | 22.2%  (8/36; 11.7–38.1) | 0.0%  (0/36; 0.0–9.6) | 0.0%  (0/36; 0.0–9.6) | S+W | MIG  2024/2025 | INS |
|  |  | *Muscicapa*  *striata* | 0.0%  (0/4; 0.0–49.0) | 0.0%  (0/4; 0.0–49.0) | 0.0%  (0/4; 0.0–49.0) | L+B | MIG  2024/2025 | INS |
|  |  | *Oenanthe*  *oenanthe* | 0.0%  (0/1; 0.0–79.3) | 0.0%  (0/1; 0.0–79.3) | 0.0%  (0/1; 0.0–79.3) | L | MIG  2025 | INS |
|  |  | *Phoenicurus*  *ochruros* | 0.0%  (0/7; 0.0–35.4) | 0.0%  (0/7; 0.0–35.4) | 0.0%  (0/7; 0.0–35.4) | R | SED  2024/2025 | INS |
|  |  | *Phoenicurus*  *phoenicurus* | 15.4%  (2/13; 4.3–42.2) | 0.0%  (0/13; 0.0–22.8) | 0.0%  (0/13; 0.0–22.8) | L+B | MIG  2024/2025 | INS |
|  |  | *Saxicola*  *rubetra* | 0.0%  (0/1; 0.0–79.3) | 0.0%  (0/1; 0.0–79.3) | 0.0%  (0/1; 0.0–79.3) | S | MIG  2025 | INS |
|  | Oriolidae | *Oriolus*  *oriolus* | 0.0%  (0/1; 0.0–79.3) | 100.0%  (1/1; 20.7–100.0) | 0.0%  (0/1; 0.0–79.3) | L+B | MIG  2024 | FRU |
|  | Panuridae | *Panurus*  *biarmicus* | 18.9%  (7/37; 9.5–34.2) | 13.5%  (5/37; 5.9–28.0) | 10.8%  (4/37; 4.3–24.7) | R | SED  2024/2025 | INS(b) + GRA(w) |
|  | Paridae | *Parus*  *caeruleus* | 4.7%  (3/64; 1.6–12.9) | 1.6%  (1/64; 0.3–8.3) | 0.0%  (0/64; 0.0–5.7) | R+P | SED  2024/2025 | INS(b) + GRA(w) |
|  |  | *Parus*  *major* | 13.6%  (24/176; 9.3–19.5) | 1.1%  (2/176; 0.3–4.0) | 4.0%  (7/176; 1.9–8.0) | R | SED  2024/2025 | INS(b) + GRA(w) |
|  | Passer | *Passer*  *domesticus* | 13.4%  (20/149; 8.9–19.8) | 5.4%  (8/149; 2.7–10.2) | 8.1%  (12/149; 4.7–13.5) | R | SED  2024/2025 | INS(b) + GRA(w) |
|  |  | *Passer*  *montanus* | 0.0%  (0/46; 0.0–7.7) | 0.0%  (0/46; 0.0–7.7) | 6.5%  (3/46; 2.2–17.5) | R | SED  2024/2025 | INS(b) + GRA(w) |
|  |  | *Petronia*  *petronia* | 0.0%  (0/4; 0.0–49.0) | 0.0%  (0/4; 0.0–49.0) | 0.0%  (0/4; 0.0–49.0) | R | SED  2024/2025 | INS(b) + GRA(w) |
|  | Phylloscopidae | *Phylloscopus*  *collybita* | 2.9%  (1/34; 0.5–14.9) | 0.0%  (0/34; 0.0–10.2) | 0.0%  (0/34; 0.0–10.2) | R+P | MIG  2024/2025 | INS |
|  |  | *Phylloscopus*  *collybita*  *tristis* | 0.0%  (0/1; 0.0–79.3) | 0.0%  (0/1; 0.0–79.3) | 0.0%  (0/1; 0.0–79.3) | L+W | MIG  2024 | INS |
|  |  | *Phylloscopus*  *trochilus* | 8.3%  (1/12; 1.5–35.4) | 16.7%  (2/12; 4.7–44.8) | 0.0%  (0/12; 0.0–24.3) | L | MIG  2024/2025 | INS |
|  | Pica | *Pica*  *pica* | 25.0%  (1/4; 4.6–69.9) | 0.0%  (0/4; 0.0–49.0) | 0.0%  (0/4; 0.0–49.0) | R | SED  2024/2025 | OMN |
|  | Regulidae | *Regulus*  *ignicapilla* | 0.0%  (0/3; 0.0–56.2) | 0.0%  (0/3; 0.0–56.2) | 0.0%  (0/3; 0.0–56.2) | R+P | SED  2025 | INS |
|  | Remizidae | *Remiz*  *pendulinus* | 0.0%  (0/18; 0.0–17.6) | 0.0%  (0/18; 0.0–17.6) | 0.0%  (0/18; 0.0–17.6) | L+W | MIG  2024/2025 | GRA |
|  | Sturnus | *Sturnus*  *vulgaris* | 3.2%  (1/31; 0.6–16.2) | 3.2%  (1/31; 0.6–16.2) | 0.0%  (0/31; 0.0–11.0) | R+P | SED  2024/2025 | OMN |
|  | Sylviidae | *Sylvia*  *atricapilla* | 4.9%  (3/61; 1.7–13.5) | 0.0%  (0/61; 0.0–5.9) | 1.6%  (1/61; 0.3–8.7) | R+P | SED  2024/2025 | INS |
|  |  | *Sylvia*  *borin* | 0.0%  (0/5; 0.0–43.4) | 0.0%  (0/5; 0.0–43.4) | 0.0%  (0/5; 0.0–43.4) | L | MIG  2025 | INS + FRU(m) |
|  |  | *Sylvia*  *cantillans* | 0.0%  (0/4; 0.0–49.0) | 0.0%  (0/4; 0.0–49.0) | 0.0%  (0/4; 0.0–49.0) | L+B | MIG  2024/2025 | INS |
|  |  | *Sylvia*  *communis* | 33.3%  (1/3; 6.1–79.2) | 0.0%  (0/3; 0.0–56.2) | 0.0%  (0/3; 0.0–56.2) | L | MIG  2024/2025 | INS |
|  |  | *Sylvia*  *melanocephala* | 6.2%  (3/48; 2.1–16.8) | 4.2%  (2/48; 1.2–14.0) | 0.0%  (0/48; 0.0–7.4) | R | SED  2024/2025 | INS |
|  | Turdidae | *Saxicola*  *rubicola* | 0.0%  (0/7; 0.0–35.4) | 0.0%  (0/7; 0.0–35.4) | 0.0%  (0/7; 0.0–35.4) | R | SED  2024/2025 | INS |
|  |  | *Turdus*  *merula* | 0.0%  (0/3; 0.0–56.2) | 0.0%  (0/3; 0.0–56.2) | 0.0%  (0/3; 0.0–56.2) | R+P | SED  2025 | OMN |
|  |  | *Turdus*  *philomelos* | 0.0%  (0/1; 0.0–79.3) | 0.0%  (0/1; 0.0–79.3) | 0.0%  (0/1; 0.0–79.3) | S+W | MIG  2024 | OMN |
| Pelecaniformes | Ardeidae | *Ixobrychus*  *minutus* | 50.0%  (2/4; 15.0–85.0) | 0.0%  (0/4; 0.0–49.0) | 0.0%  (0/4; 0.0–49.0) | L+B | MIG  2024 | PIS |
| Piciformes | Picidae | *Dryobates*  *minor* | 0.0%  (0/1; 0.0–79.3) | 0.0%  (0/1; 0.0–79.3) | 0.0%  (0/1; 0.0–79.3) | R | SED  2025 | INS |
|  |  | *Jynx*  *torquilla* | 0.0%  (0/4; 0.0–49.0) | 0.0%  (0/4; 0.0–49.0) | 0.0%  (0/4; 0.0–49.0) | S+W | MIG  2024/2025 | INS |
| Strigiformes | Strigidae | *Strix*  *aluco* | 0.0%  (0/3; 0.0–56.2) | 33.3%  (1/3; 6.1–79.2) | 0.0%  (0/3; 0.0–56.2) | R | SED  2025 | CAR |
|  | Tytonidae | *Tyto*  *alba* | 6.5%  (3/46; 2.2–17.5) | 2.2%  (1/46; 0.4–11.3) | 0.0%  (0/46; 0.0–7.7) | R | SED  2024/2025 | CAR |

**Supplementary Table 1:** Summary of species traits and prevalence. The table presents species-level information including taxonomic identity, prevalence values, ecological traits, and associated classifications. For each species, prevalence represents the proportion of positive observations relative to the total number of individuals sampled, expressed as n/N with 95% confidence intervals (95% CI). USUV and WNV prevalences correspond to single infections only, whereas co-infection refers exclusively to simultaneous detection of both viruses in the same individual. Migration status is coded as follows: R = Resident; P = Partial migrant; S = Short-distance migrant; L = Long-distance migrant; W = Wintering; B = Breeding. SED/MIG 2024/2025 indicates the study-level classification of species as sedentary (SED) or migratory (MIG), and the year(s) of sampling (2024, 2025, or both). Diet category is coded as follows: INS = Insectivorous; INV = Invertivorous; OMN = Omnivorous; GRA = Granivorous; FRU = Frugivorous; CAR = Carnivorous; PIS = Piscivorous; m = migration; b = breeding; w = winter.
