# Supplementary material for: Widespread circulation of West Nile and Usutu viruses in sedentary and migratory avifauna: A two-year study (2024–2025) of active surveillance in South of France
Source: PLoS Negl Trop Dis. 2026 Sep 3;20(9):e0014397. doi: 10.1371/journal.pntd.0014397 (PMC13568517; doi:10.1371/journal.pntd.0014397)
Supplement: Supplementary file 2 [file pntd.0014397.s002.docx]

| **Virus** | **Variable** | **Category** | **Positive, n (%)** | **Negative, n (%)** | **Total** | |
| --- | --- | --- | --- | --- | --- | --- |
| USUV | Migration pattern | Sedentary | 140 (13.3%) | 909 (86.7%) | 1049 | 2533 |
|  |  | Migratory | 163 (11.0%) | 1321 (89.0%) | 1484 |  |
|  | Order | Non-Passeriformes | 22 (9.0%) | 223 (91.0%) | 245 | 2533 |
|  |  | Passeriformes | 281 (12.3%) | 2007 (87.7%) | 2288 |  |
|  | Year | 2024 | 144 (14.1%) | 877 (85.9%) | 1021 | 2533 |
|  |  | 2025 | 159 (10.5%) | 1353 (89.5%) | 1512 |  |
|  | Season | Summer | 186 (14.4%) | 1107 (85.6%) | 1293 | 2533 |
|  |  | Autumn/Winter | 31 (5.8%) | 499 (94.2%) | 530 |  |
|  |  | Spring | 86 (12.1%) | 624 (87.9%) | 710 |  |
|  | Locality | Rural | 289 (12.7%) | 1979 (87.3%) | 2268 | 2533 |
|  |  | Urban/Peri-urban | 14 (5.3%) | 251 (94.7%) | 265 |  |
|  | Age | 1A | 215 (10.9%) | 1752 (89.1%) | 1967 | 2530 |
|  |  | 2A+ | 34 (11.5%) | 262 (88.5%) | 296 |  |
|  |  | PUL | 28 (18.3%) | 125 (81.7%) | 153 |  |
|  |  | VOL | 26 (22.8%) | 88 (77.2%) | 114 |  |
|  | Sex | Female | 49 (10.4%) | 422 (89.6%) | 471 | 1045 |
|  |  | Male | 59 (10.3%) | 515 (89.7%) | 574 |  |
|  | Feeding | Insectivorous | 194 (12.0%) | 1427 (88.0%) | 1621 | 2533 |
|  |  | Insectivorous breeding / granivorous winter | 101 (13.2%) | 664 (86.8%) | 765 |  |
|  |  | Insectivorous / frugivorous migration | 0 (0.0%) | 5 (100.0%) | 5 |  |
|  |  | Invertivorous | 0 (0.0%) | 2 (100.0%) | 2 |  |
|  |  | Granivorous | 0 (0.0%) | 18 (100.0%) | 18 |  |
|  |  | Frugivorous | 0 (0.0%) | 1 (100.0%) | 1 |  |
|  |  | Carnivorous | 3 (5.8%) | 49 (94.2%) | 52 |  |
|  |  | Omnivorous | 2 (4.9%) | 39 (95.1%) | 41 |  |
|  |  | Piscivorous | 3 (10.7%) | 25 (89.3%) | 28 |  |
| WNV | Migration pattern | Sedentary | 60 (5.7%) | 989 (94.3%) | 1049 | 2533 |
|  |  | Migratory | 50 (3.4%) | 1434 (96.6%) | 1484 |  |
|  | Order | Non-Passeriformes | 20 (8.2%) | 225 (91.8%) | 245 | 2533 |
|  |  | Passeriformes | 90 (3.9%) | 2198 (96.1%) | 2288 |  |
|  | Year | 2024 | 79 (7.7%) | 942 (92.3%) | 1021 | 2533 |
|  |  | 2025 | 31 (2.1%) | 1481 (97.9%) | 1512 |  |
|  | Season | Summer | 31 (2.4%) | 1262 (97.6%) | 1293 | 2533 |
|  |  | Autumn/Winter | 2 (0.4%) | 528 (99.6%) | 530 |  |
|  |  | Spring | 77 (10.8%) | 633 (89.2%) | 710 |  |
|  | Locality | Rural | 107 (4.7%) | 2161 (95.3%) | 2268 | 2533 |
|  |  | Urban/Peri-urban | 3 (1.1%) | 262 (98.9%) | 265 |  |
|  | Age | 1A | 59 (3.0%) | 1908 (97.0%) | 1967 | 2530 |
|  |  | 2A+ | 19 (6.4%) | 277 (93.6%) | 296 |  |
|  |  | PUL | 27 (17.6%) | 126 (82.4%) | 153 |  |
|  |  | VOL | 4 (3.5%) | 110 (96.5%) | 114 |  |
|  | Sex | Female | 19 (4.0%) | 452 (96.0%) | 471 | 1045 |
|  |  | Male | 35 (6.1%) | 539 (93.9%) | 574 |  |
|  | Feeding | Insectivorous | 58 (3.6%) | 1563 (96.4%) | 1621 | 2533 |
|  |  | Insectivorous breeding / granivorous winter | 48 (6.3%) | 717 (93.7%) | 765 |  |
|  |  | Insectivorous / frugivorous migration | 0 (0.0%) | 5 (100.0%) | 5 |  |
|  |  | Invertivorous | 0 (0.0%) | 2 (100.0%) | 2 |  |
|  |  | Granivorous | 0 (0.0%) | 18 (100.0%) | 18 |  |
|  |  | Frugivorous | 1 (100.0%) | 0 (0.0%) | 1 |  |
|  |  | Carnivorous | 2 (3.8%) | 50 (96.2%) | 52 |  |
|  |  | Omnivorous | 1 (2.4%) | 40 (97.6%) | 41 |  |
|  |  | Piscivorous | 0 (0.0%) | 28 (100.0%) | 28 |  |

**Supplementary Table 2:** Distribution of sampled birds across the categories considered in the analyses. The table presents the number of individuals in each category according to year, season, locality type, age class, sex, migratory status, taxonomic group, and feeding guild. Sex was undetermined for 1488 individuals, and age information was missing for three individuals for both WNV and USUV datasets.
